# Bradyrhizobium tunisiense sp. nov., a novel rhizobial species isolated from Acacia saligna nodules

**DOI:** 10.1099/ijsem.0.006807

**Published:** 2025-06-19

**Authors:** Jihed Hsouna, Houda Zouagui, Takwa Gritli, Houda Ilahi, Jia-Cheng Han, Muhammad Sulman, Walid Ellouze, Xiao Xia Zhang, Maroua Mansouri, Mustapha Missbah El Idrissi, Soufiane Alami, Pierre Emmanuel Courty, Daniel Wipf, Abdelkader Bekki, James T. Tambong, Bacem Mnasri

**Affiliations:** 1 Laboratory of Legumes and Sustainable Agroecosystems, Centre of Biotechnology of Borj-Cédria, BP 901, Hammam-Lif 2050, Tunisia; 2 Faculty of Sciences, Laboratory of Microbiology and Molecular Biology, Mohammed V University in Rabat, Rabat, Morocco; 3 Agricultural Cultural Collection of China, Institute of Agricultural Resources and Regional Planning, Chinese Academy of Agricultural Sciences, Beijing 100080, PR China; 4 Agriculture and Agri-Food Canada, 4902 Victoria Avenue North, Vineland Station, Ontario, L0R 2E0, Canada; 5 Faculty of Sciences, Centre de Biotechnologies Végétale et Microbienne, Biodiversité et Environnement, Mohammed V University in Rabat, Rabat, Morocco; 6 Agroécologie, Institut Agro Dijon, CNRS, Univ. Bourgogne, INRAE, Univ. Bourgogne Franche-Comté, F-21000 Dijon, France; 7 Department of Biotechnology, Faculty of Sciences, Biotechnology of Rhizobia and Plant Breeding Laboratory, University of Oran1, Sénia, Algeria; 8 Agriculture and Agri-Food Canada, 960 Carling Avenue, Ottawa, Ontario, K1A 0C6, Canada

**Keywords:** *Acacia saligna*, *Bradyrhizobium*, phylogenomics, rhizobia, whole genomes

## Abstract

Three slow-growing rhizobial strains, designated as 1AS2L^T^, 1AS20L and 1AS5L, were isolated from nodules of *Acacia saligna* in Borj Cedria, northern Tunisia. These strains, which belong to the symbiovar cyanophyllae, were characterized using a polyphasic approach. Phylogenetic analysis of the 16S rRNA (*rrs*) gene placed these strains within the genus *Bradyrhizobium*, specifically in the superclade associated with *Bradyrhizobium japonicum*. Further phylogenetic analysis using concatenated sequences of the *rec*A, *atp*D, *gln*II and *gyr*B genes (totalling 1,734 bp) positioned the strains in a distinct lineage, with *Bradyrhizobium shewense* identified as their closest related species, sharing a sequence identity of 95.2%. The type strain, 1AS2L^T^, exhibited average nucleotide identity values of 89.10%, 89.08% and 89.00% with the type strains of the closest valid species: *Bradyrhizobium frederickii*, *B. shewense* and *Bradyrhizobium ottawaense*, respectively. Additionally, digital DNA–DNA hybridization values confirmed the novelty of strain 1AS2L^T^, showing low similarity (38.0%–38.3%) with the type strains of the closest known species. Phylogenomic analyses based on up-to-date bacterial core genes, Type (Strain) Genome Server and the Genome Taxonomy Database (GTDB) pipelines further supported the uniqueness of the 1AS2L^T^, 1AS20L and 1AS5L strains. The GTDB analysis also robustly clustered two strains (SZCCT0449 and NSD-1) with our strains, suggesting putative members of the proposed novel species. The differentiation of these novel strains from their closest phylogenetic neighbours was also corroborated by phenotypic, physiological and fatty acid content analyses. Based on genomic, phenotypic and biochemical data, we propose the establishment of a novel species, *Bradyrhizobium tunisiense* sp. nov., with strain 1AS2L^T^ (=LMG 33170^T^=DSM 114401^T^) as the type strain.

## Introduction

Legumes possess a unique ability to biologically fix nitrogen through a symbiotic relationship with rhizobia, which induce the formation of nodules on their roots. These nodules create an optimal microhabitat for the bacteria, providing them with carbon compounds derived from the plant’s photosynthesis. In return, the bacteria fix atmospheric nitrogen and supply it to the plant in the form of ammonia (NH₃). The genus *Bradyrhizobium* englobes one of the most efficient nitrogen fixers and is considered to be the most diverse genus among rhizobia, although this diversity is not fully reflected in the number of described species [1]. The genus *Bradyrhizobium* was first recognized as distinct in 1982, with *Bradyrhizobium japonicum* being the first species described [2]. Since then, the number of recognized *Bradyrhizobium* species has significantly increased, and the genus now comprises 73 validly published species and 2 subspecies ([3]; accessed on 5 December 2024; https://lpsn.dsmz.de/search?word=bradyrhizobium).

*Bradyrhizobium* species are known for their ability to form symbiotic nodules with a variety of legume species. They are reported to establish effective nodules with important crop legumes such as common bean [4], soybean [5] and peanut [6], as well as leguminous trees like *Acacia* and *Leucaena* [7,8], and various spontaneous legume species [9].

For species identification within *Bradyrhizobium*, the *rrs* gene and a set of housekeeping genes, including *rec*A, *atp*D, *gyr*B and *gln*II, are commonly used [7,10]. Currently, genomes for 1,335 *Bradyrhizobium* strains are publicly available and are routinely used to delineate members of the genus. *Bradyrhizobium* species typically possess large genomes with few plasmids and, with rare exceptions, lack symbiosis plasmids [11]. Recent advances in whole-genome sequencing have introduced new metrics for species delineation, such as average nucleotide identity (ANI) calculations [12] and phylogenomics [13].

To study symbiotic diversity, markers such as *nif* and *nod* genes are frequently used in phylogenetic analyses. Significant diversity exists in the *nod*A and *nod*C genes [14,15], with phylogenetic studies revealing evidence of both vertical and horizontal gene transfer among *Bradyrhizobium* strains [7,16]. Symbiovars have been identified for certain *Bradyrhizobium* species [17], and new symbiovars are continually being described [7,18,23].

In Tunisia, *Bradyrhizobium* strains have been isolated from various legume species, including *Lupinus* [24,25], *Arachis* [26] and *Genista* [27]. Recent studies by Hsouna *et al*. [7] observed that *Acacia saligna* exhibits a high degree of nodulation promiscuity, being predominantly nodulated by slow-growing bacteria which could be new species within the genus *Bradyrhizobium*. Given the increasing emphasis on whole-genome sequence analysis for describing new bacterial species [28], strains 1AS20L, 1AS2L and 1AS5L were thoroughly investigated using whole-genome sequencing and phenotypic tests in the present study. Based on these polyphasic data and analyses, a new species, *Bradyrhizobium tunisiense* sp. nov., is proposed.

## Methods

### Isolation and ecology

Three novel *Bradyrhizobium* strains, 1AS2L^T^, 1AS5L and 1AS20L, were isolated from surface-sterilized root nodules of *A. saligna* grown in Borj Cedria (36° 42′ 35.5″ N 10° 25′ 42″ E), located in northern Tunisia, following the method described by Vincent [29]. The strains were cultured on yeast mannitol agar (YMA) at 28 °C for 6 days. Single colonies were then picked and verified for purity by repeated streaking as described by Hsouna *et al*. [7]. These novel strains, 1AS2L^T^, 1AS5L and 1AS20L, were previously classified as symbiovar cyanophyllae [7]. The type strain, 1AS2LT, has been deposited as LMG 33170, in the BCCM/LMG Bacteria Collection at the University of Ghent, Belgium, and as DSMZ 114401 in the DSMZ-German Collection of Microorganisms and Cell Cultures, Leibniz Institute.

### 16S rRNA and *atp*D-*rec*A-*gln*II-*gyr*B concatenated phylogenetic analyses

For phylogenetic analyses, sequences of the 16S rRNA and concatenated *atp*D, *gln*II, *gyr*B and *rec*A core genes were utilized. DNA was extracted from *Bradyrhizobium* strains 1AS2L^T^, 1AS5L and 1AS20L cultured in tryptone-yeast medium [30], following the method described by Terefework *et al*. [31]. The 16S rRNA gene sequence (1,230 bp) and partial sequences of the housekeeping genes (*atp*D, *rec*A, *gln*II and *gyr*B) and the symbiotic gene (*nod*C) were amplified using standard PCR protocols. Primers used for amplification were those described by Hsouna *et al*. [7]. PCR products were purified with the GeneJET Gel Extraction Kit (Thermo Scientific) according to the manufacturer’s instructions. The purified DNA fragments were sequenced at the Genome Quebec Innovation Centre, Montreal, Canada.

The sequences of the 16S rRNA gene, the concatenated housekeeping genes (*atp*D, *rec*A, *gln*II and *gyr*B) and the symbiotic gene (*nod*C) were employed for phylogenetic analyses. Sequence similarities were assessed using the blast programme (http://blast.ncbi.nlm.nih.gov/Blast.cgi), and reference sequences were retrieved from GenBank (NCBI). Sequence alignment was performed using ClustalW2 (http://www.ebi.ac.uk/Tools/clustalw2/). Phylogenetic trees were constructed based on the 16S rRNA, *nod*C gene or *atp*D-*rec*A-*gln*II-*gyr*B concatenated pseudomolecules using the maximum-likelihood (ML) method implemented in mega 7.0 [32]. The Kimura two-parameter model was used for evolutionary analysis [33], and bootstrap support was assessed with 1,000 replicates.

### Genomic relatedness and phylogenomics

The draft whole-genome sequences of the three novel strains were generated using Illumina NovaSeq 6000 technology by Génome Québec in Montreal, Canada. To ensure data integrity, we conducted a quality assessment of the raw reads (2×151 bp) with FastQC software [34]. *De novo* genome assemblies were then created using Unicycler v0.4.8 [35], and their quality was evaluated with QUAST [36], integrated into PATRIC 3.6.12 or Bacterial and Viral Bioinformatics Resource Center (BV-BRC 3.42.3) [37,39]. Contigs shorter than 300 bp were excluded from the analysis. The assembled contigs were annotated using GeneMarkS-2+ [40] as implemented in with the NCBI Prokaryotic Genome Annotation Pipeline [41]. Basic statistics summarizing the features of the assembled draft genomes are provided in Table 1.

**Table 1.** Basic statistic of whole genomes of the three strains of *B. tunisiense* sp. nov., sequenced using Illumina NovaSeq 6000

Description	1AS2L^T^	1AS20L	1AS5L
GenBank accession number	JBHLEX01	JBHLEV01	JBHLEW01
Number of short paired-end reads (151 bp)	33,112,583	33,112,583	33,112,583
Average coverage depth	1,238.9×	1,273.3×	1,193.4×
Contig count	49	26	60
Largest contig (bp)	1,126,891	1,600,489	1,285,818
Total length (bp)	7,928,546	7,716,925	8,266,930
Contigs N_50_ (bp)	853,907	1,149,130	512,106
Contigs L_50_	5	3	6
Total number of genes	7,432	7,264	7,855
CDSs with	3,633	7,105	7,649
Total CDSs	7,363	7,206	7,649
ncRNAs	3	4	4
rRNA: 5S, 16S, 23S	1, 1, 1	1, 1, 1	1, 1, 1
Number of tRNA	52	51	51
G+C content (mol%)	63.9	64.0	63.7

To verify the taxonomic status of strains 1AS2L^T^, 1AS5L and 1AS20L, ANI values were estimated using the OrthoANIu tool v1.2 [42], and DNA–DNA hybridization (dDDH) values were calculated using the Genome-to-Genome Distance Calculator v3.0 [43,44]. Up-to-date bacteria core gene version 2 (UBCG2 [45]) and Type (Strain) Genome Server (TYGS [43,46]) pipelines were used to infer phylogenomic relatedness of the three strains to known *Bradyrhizobium* species. Additionally, ANI and dDDH calculations were performed between 1AS2L^T^ and 225 metagenome-assembled genomes (MAGs) available in GenBank as of 5 August 2024.

In addition, a comprehensive taxonomic classification of isolates 1AS2L^T^, 1AS5L and 1AS20L was conducted using all genome sequences from the most recent GTDB release (09-RS220) [47] with the GTDB-Tk v2.4.0 pipeline [48]. The pipeline initially compared the strains against GTDB representative genomes based on ANI estimates calculated with skani v0.2.2 [49]. Gene prediction was performed using Prodigal v2.6.3 [50], and homologues of 120 bacterial gene markers were identified using HMMER v3.4 [51]. The ML placement of the isolates in the GTDB-Tk reference tree was determined with pplacer v1.1 [52]. FastTree v2.1.11 [53] was used to infer a *de novo* approximately ML tree for the genus *Bradyrhizobium*. This tree was constructed from a multiple sequence alignment of 117 bacterial gene markers (Table S2, available in the online Supplementary Material) [47] using the LG amino acid substitution model [54] and was rooted with representatives of the *Nitrobacter* genus.

### Physiology and chemotaxonomy

Various phenotypic and biochemical properties of the three novel strains were compared with closely related *Bradyrhizobium* species. Colony morphology was assessed on yeast ectract mannitol agar (YMA) plates incubated at 28 °C for 5–7 days. Growth conditions were tested in liquid yeast extract mannitol (YEM) broth at different temperatures (4 °C, 20 °C, 28°C and 37 °C), at varying NaCl concentrations [0.2%, 0.4%, 0.6% and 0.8% (w/v)] and across a pH range of 5.0–10.0 (in increments of 1.0 pH unit). These tests were carried out by incubation at 28 °C for 5–7 days unless otherwise specified. Antibiotic resistance was evaluated on YEM plates supplemented with five concentrations of each antibiotic (kanamycin, neomycin, ampicillin, erythromycin and penicillin) at 5, 10, 20, 50 and 80 µg ml^−1^. All tests were conducted in triplicate. Additionally, phenotypic tests, including carbon-source utilization and chemical sensitivity assays, were performed using Biolog GEN III MicroPlates following the manufacturer’s instructions. Bacterial suspensions with a transmittance of 95%–98% at 590 nm were inoculated into the GN3 plates and incubated at 28 °C for 72 h. Positive utilization of carbon sources was indicated by a purple coloration and measured spectrophotometrically using a Biolog standardized instrument. Data were statistically analysed by calculating mean absorbance values obtained from three independent biological replicates.

Enzymatic activities were analysed with API ZYM system strips (bioMérieux, France). The fatty acid composition was analysed using the MIDI Sherlock Microbial Identification System version 6.1 and the TSBA6 database. Bacterial biomass was obtained from cells grown on yeast extract mannitol agar (YMA) plates incubated at 28 °C for 5–7 days. The harvested biomass was then processed according to the standard protocol recommended by the MIDI Sherlock Microbial Identification System.

## Results and discussion

### Phylogenetic analyses

ML phylogenetic analysis of partial 16S rRNA sequences (1,230 bp) confirmed that the strains 1AS2L^T^, 1AS5L and 1AS20L examined in this study belong to the superclade associated with *B. japonicum* within the genus *Bradyrhizobium* (Fig. S1). The type strain 1AS2L^T^ exhibited 100% sequence homology to *Bradyrhizobium nitroreducens* TSA1^T^, a bacterium previously isolated from rice paddy soil in Japan [55]. Additionally, the novel strains show 99.92% similarity with *Bradyrhizobium frederickii* CNPSo 3426^T^, isolated from nodules of the caesalpinioid species *Chamaecrista fasciculata* [56]; 99.84% similarity with *Bradyrhizobium nannigensis* CCBAU53390^T^, a bacterium from effective nodules of peanut [57]; and 99.84% similarity with *Bradyrhizobium centrosemae* A9^T^, isolated from root nodules of *Centrosema* species native to America [20]. These results confirm previous reports that the 16S rRNA gene is a reliable genus-level marker but has serious limitations for species delineation within the genus *Bradyrhizobium* [7,58].

MLSA of core gene sequences is a more reliable method for phylogenetic analysis and species delineation within the genus *Bradyrhizobium* [59,60]. ML phylogenetic analysis of the *rec*A-*gln*II-*atp*D-*gyr*B concatenated housekeeping genes clearly distinguished the three novel strains from all known *Bradyrhizobium* type strains (Fig. S2). Strains 1AS2L^T^, 1AS20L and 1AS5L exhibited 100% similarity to each other and formed a distinct clade with a high bootstrap value (100%). However, strain 1AS2L^T^ showed only 95.2% and 94.8% similarity to the closest type strains, *Bradyrhizobium shewense* ERR11^T^ and *Bradyrhizobium ottawaense* OO99^T^, respectively (Fig. S2). The type strain *B. shewense* ERR11^T^ was isolated from root nodules of the leguminous tree, *Erythrina brucei*, native to Ethiopia [61], while *B. ottawaense* OO99^T^ was isolated from root nodules of *Glycine max* in Ottawa, Canada [62]. These results, particularly the unique positioning in the MLSA tree (Fig. S2), supported the proposal that these three strains represent a putative novel species within the genus *Bradyrhizobium*.

### Genomic relatedness and phylogenomics

Table 1 presents the basic statistics of the assembled draft genomes for strains 1AS2L^T^, 1AS20L and 1AS5L. Each strain produced slightly over 30 million raw NovaSeq reads. Following quality checks, the reads used in the assembly process resulted in average coverage depths of 1,238×, 1,273× and 1,193×, with contig counts of 49, 26 and 60 for strains 1AS2L^T^, 1AS20L and 1AS5L, respectively. The genome sequences were 99.6% complete, with coarse and fine consistencies of 99.4%, 99.9% and 96.7%, and a contamination rate ranging from 3.1% to 4.2%.

The N_50_ values were 853,907, 1,149,130 and 512,106 bp, with L50 values of 5, 3 and 6 for strains 1AS2L^T^, 1AS20L and 1AS5L, respectively. The total lengths of the draft genomes were 7,928, 546,702, 7,716,925 and 8,266,930 bp for strains 1AS2L^T^, 1AS20L and 1AS5L, respectively, with G+C contents of 63.7–64.0 mol% (Table 1).

A total of 7,432 genes were identified in 1AS2L^T^, 7,264 in 1AS20L and 7,855 in 1AS5L, and CDSs with protein numbered 7,268, 7,105 or 7,649, respectively. Additionally, 51 tRNAs were identified in strains 1AS20L^T^ and 1AS5L, while 52 tRNAs were found in strain 1AS2L. Each strain also contained three rRNAs (5S, 16S and 23S) and three or four noncoding RNAs (Table 1).

Genome-based dDDH and ANI analyses were performed against several closely related type strains and unidentified species within the genus *Bradyrhizobium* to confirm the taxonomic placement of the studied strains (Table 2). Strain 1AS2L^T^ exhibited dDDH and ANI values of 88.2%–87.4% and 98.55%–98.46% with strains 1AS5L and 1AS20L, all values above the 70% and 96% species-level thresholds, respectively (Table 2). However, all the 17 closely related and validly published *Bradyrhizobium* species had dDDH (30.8%–38.3%) and ANI (86.86%–90.15%) values that are lower than the above-indicated species-level delineation thresholds (Table 2).

**Table 2.** ANI and dDDH values between the type strain of *B. tunisiense* sp. nov. 1AS2L^T^ and related *Bradyrhizobium* species. (model CI), CI of the model used; Prob. dDDH>=70%, the probability that the dDDH value is >=70%

*Bradyrhizobium* species	ANI (%)	dDDH (model CI) (%)	Prob. dDDH>=70%	Distance
***B. tunisiense* 1AS2L^T^ (JBHLEX01)**	**100**	**100**	**98.30**	**0.000**
***B. tunisiense* 1AS20L (JBHLEV01)**	**98.55**	**88.2 (85.7–90.3)**	**95.13**	**0.014**
***B. tunisiense* 1AS5L (BHLEW01)**	**98.46**	**87.4 (84.8–89.6)**	**94.82**	**0.015**
*B. frederickii* CNPSo 3426^T^ (SPQS01)	90.15	38.3 (35.8–40.8)	1.74	0.105
*B. ottawaense* OO99^T^(CP029425)	89.95	38.0 (35.6–40.6)	1.62	0.106
*B. nanningense* CCBAU 53390T (LBJC01)	89.91	37.9 (35.4–40.4)	1.55	0.107
*B. shewense* ERR11^T^ (FMAI01)	89.87	38.0 (35.5–40.5)	1.60	0.106
*B. symbiodeficiens* 101S1MB^T^ (CP050066)	89.76	37.2 (34.8–39.8)	1.29	0.109
*B. nitroreducens* TSA1^T^ (LFJC01)	89.75	37.2 (34.7–39.7)	1.28	0.109
*B. amphicarpaeae* 31S1MB^T^ (CP029426)	89.63	36.9 (34.4–39.4)	1.17	0.111
*B. liaoningense* NBRC 100396^T^ (BSOX01)	89.01	35.8 (33.4–38.3)	0.84	0.115
*B. niftali* CNPSo 3448^T^ (SPQT01)	88.78	34.8 (32.3–37.3)	0.61	0.119
*B. diazoefficiens* USDA 110^T^ (CP011360)	88.59	34.6 (32.2–37.1)	0.58	0.120
*B. stylosanthis* BE 446T (LVEM01)	88.48	34.0 (31.6–36.5)	0.47	0.123
*B. arachidis* CCBAU 05117^T^ (CP030050)	88.32	33.8 (31.3–36.3)	0.44	0.124
*B. japonicum* USDA 6^T^ (AP012206)	88.26	34.1 (31.7–36.6)	0.49	0.122
*B. betae* CECT 5829^T^ (JANTYR01)	88.13	33.4 (30.9–35.9)	0.38	0.125
*B. cosmicum* S23321^T^ (CP041656)	88.03	33.0 (30.6–35.5)	0.33	0.127
*B. rifense* CTAW71^T^ (VSSS01)	87.15	31.6 (29.2–34.1)	0.20	0.134
*B. guangdongense* CCBAU 51649^T^ (CP030051)	86.86	30.8 (28.4–33.3)	0.14	0.138

**Fig. 1.**
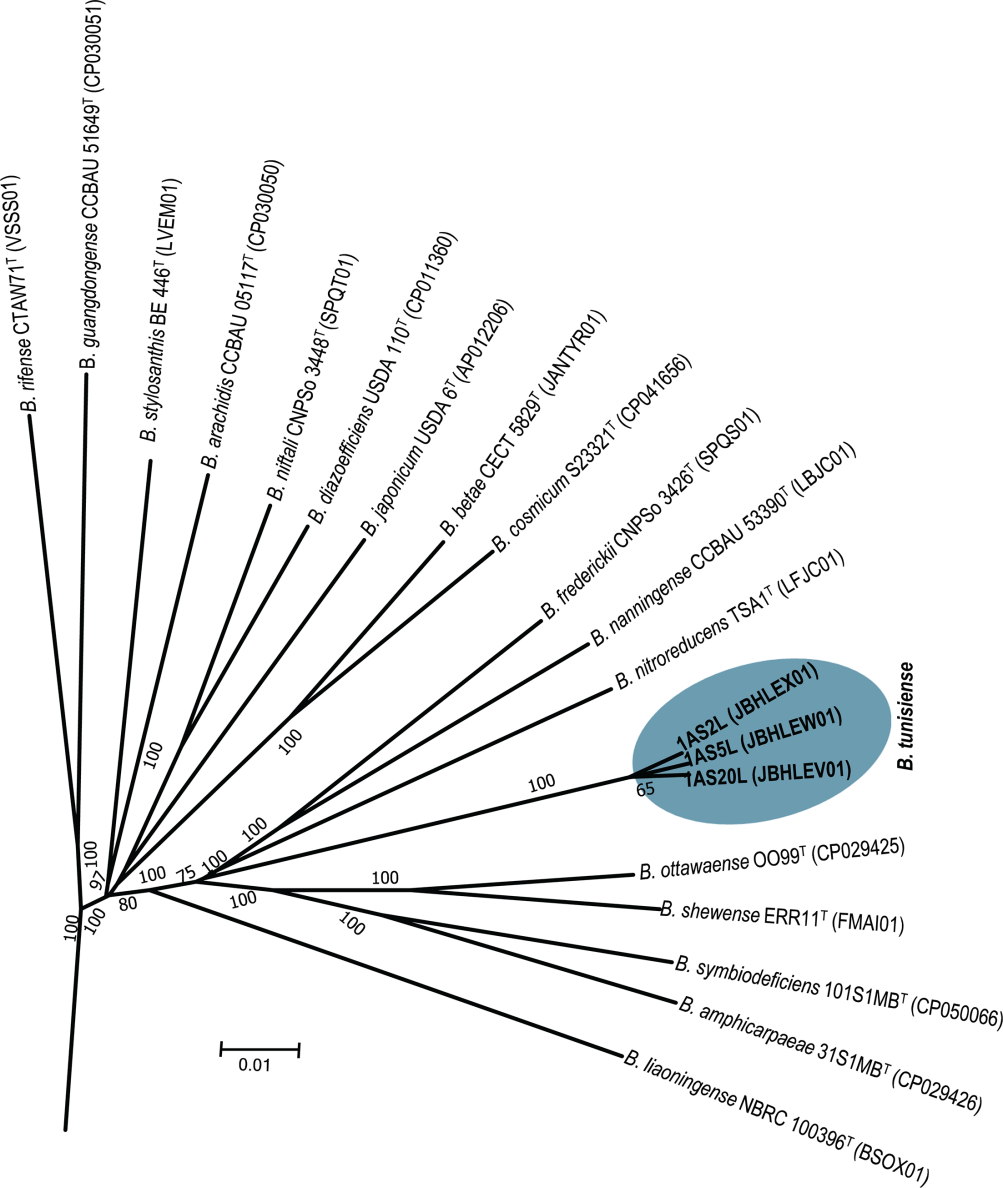
Phylogenomic tree inferred based on the Genome blast Distance Phylogeny (GBDP) method using whole-genome sequences of *B. tunisiense* strains and closely related species. Tree generated using FastME 2.1.6.1 as implemented with the TYGS pipeline. The branch lengths are scaled in terms of the GBDP distance formula d5. The numbers above branches are GBDP pseudo-bootstrap support values >50% from 100 replications, with an average branch support of 91.6%. The tree was rooted at the midpoint.

**Fig. 2.**
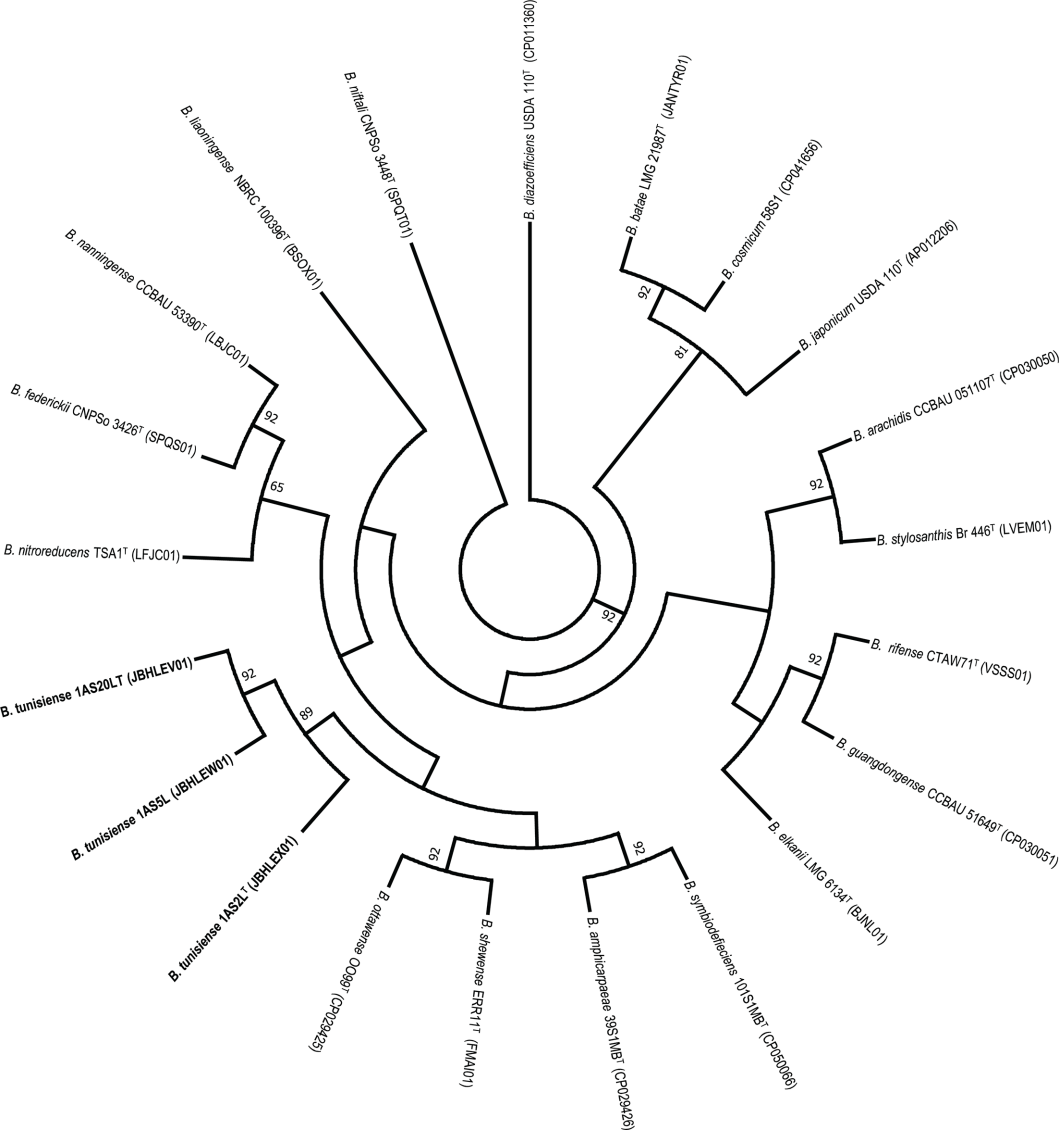
Strains of *B. tunisiense* sp. nov. clustered uniquely (bold) on the ML-based phylogenetic tree generated using the UBCG2 pipeline. The tree was inferred using RAxML-NG with the GTR+CAT model, and percentage bootstrap values are given at branching points.

Among the closest relatives, *B. frederickii* CNPso 3426^T^ had the highest dDDH estimate at 38.3% and an ANI value of 90.15% with strain 1AS2L^T^. *B. ottawaense* OO99^T^ and *B. shewense* ERR11^T^ followed closely, with dDDH and ANI values of 38% and ~89%, respectively (Table 2). These results corroborate the findings from the *rec*A-*atp*D-*gyr*B-*gln*II phylogenetic analysis, suggesting that strains 1AS2L^T^, 1AS20L and 1AS5L represent a novel species within the genus *Bradyrhizobium*. The TYGS (Fig. 1) and UBCG2 (Fig. 2) trees also provide insights into the relationships between *B. tunisiense* and other *Bradyrhizobium* species.

**Fig. 3.**
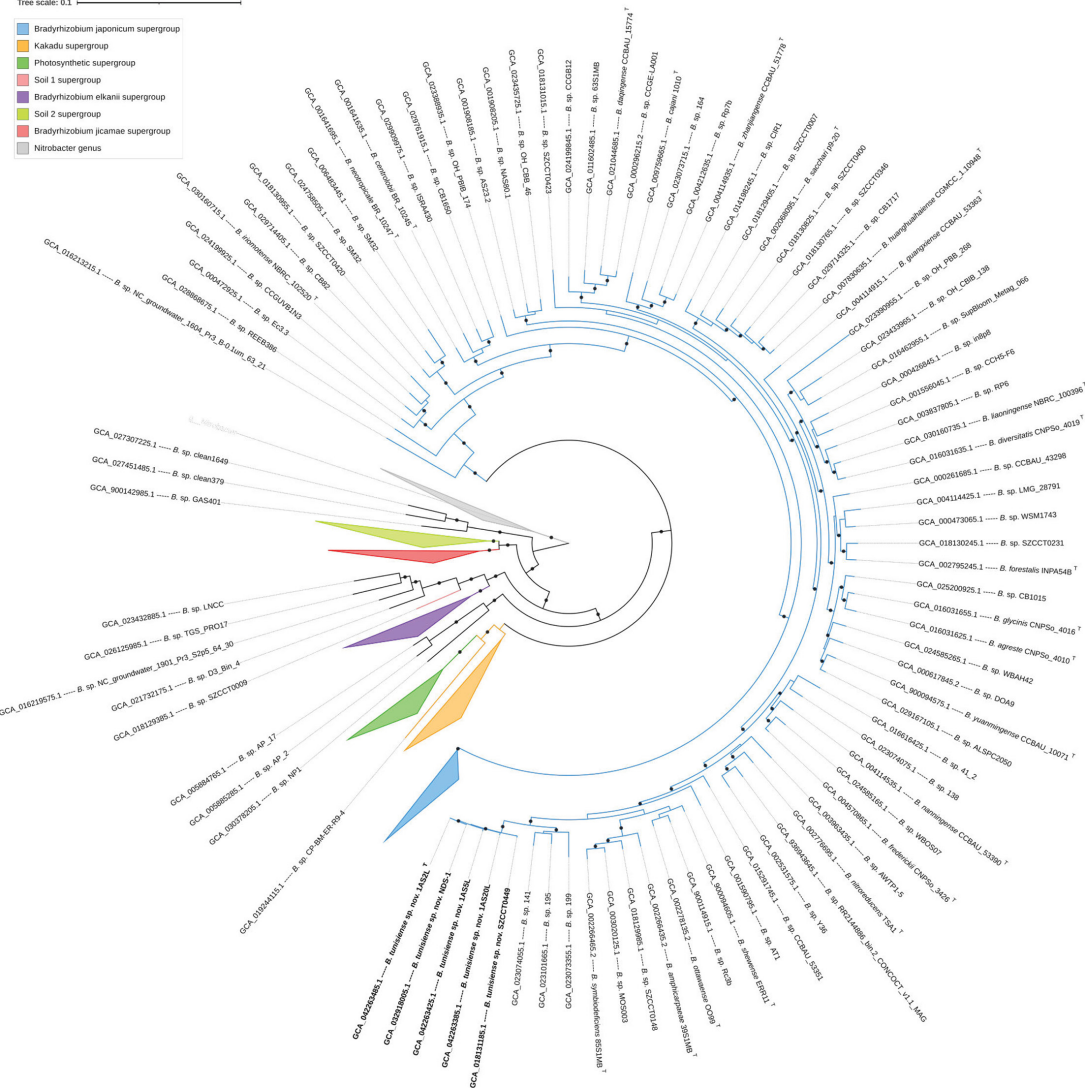
*De novo* approximately ML phylogenomic tree of the genus *Bradyrhizobium*, with the novel strains highlighted in bold. The tree was constructed using 117 bacterial gene markers and GTDB reference genomes and was rooted to the genus *Nitrobacter*. Local support values ≥70 are shown as circles on the corresponding branches (based on 1,000 resamples). Type strains are indicated by a superscript ‘T’. An ANI threshold of 96% was used for strain identification. The scale bar represents the number of substitutions per site.

Furthermore, comparisons between strain 1AS2L^T^ and 225 MAGs revealed that all ANI and dDDH estimates were below the standard thresholds for species delineation (Table S1). Phylogenomic analysis using the GTDB analysis based on the ANI values and ML placement consistently classified the strains 1AS2L^T^, 1AS5L and 1AS20L within the genus *Bradyrhizobium* (Fig. 3) but did not assign them to any validly published species (Fig. 2). The *de novo* phylogenomic tree of the *Bradyrhizobium* genus (Fig. S3) demonstrated that most reference genomes in the database clustered into seven previously described supergroups [63]. Strains 1AS2L^T^, 1AS5L and 1AS20L were positioned within the *B. japonicum* supergroup (Fig. S3).

**Fig. 4.**
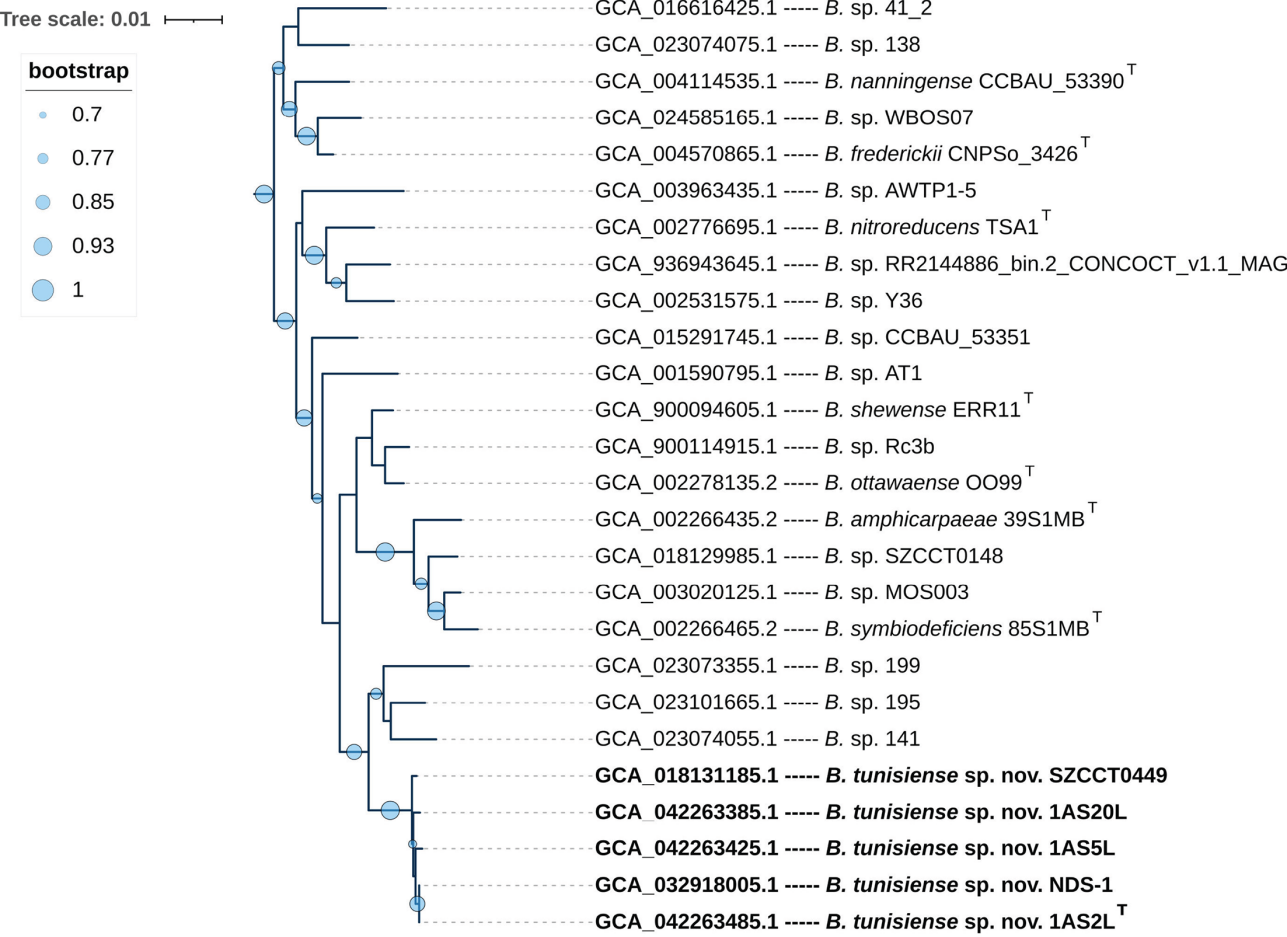
Subtree from the phylogenomic tree of the genus *Bradyrhizobium*. The three novel strains are highlighted in bold. This subtree is derived from the *B. japonicum* supergroup. Local support values of ≥70 are indicated by circles on the corresponding branches (based on 1,000 resamples). Type strains are marked with a superscript ‘T’. An ANI threshold of 96% was used to identify the strains. The scale bar represents the number of substitutions per site.

Additionally, the GTDB analysis clustered our three isolates with two NCBI entries: a Tunisian strain, *Bradyrhizobium* sp. NDS-1 (GCA_032918005.1), and a Chinese isolate, SZCCT0449 (GCA_018131185.1), deposited in GenBank as *Bradyrhizobium diazoefficiens*, suggesting that these are the closest taxonomic relatives to the studied strains. For a more detailed examination of the phylogenetic position of the three strains, a subtree was extracted from the *B. japonicum* supergroup (Fig. 4). This subtree revealed that strains 1AS2L^T^, 1AS5L and 1AS20L, along with *Bradyrhizobium* sp. NDS-1 and *B. diazoefficiens* SZCCT0449, formed a highly supported monophyletic cluster, corresponding to the proposed novel species (Fig. 4). Strain 1AS2L^T^ exhibited ANI or dDDH values of 99.60% and 98.76% or 99.00% and 89.00% with strains NDS-1 and SZCCT0449, respectively. Strain NDS-1 was isolated from the root nodule of peanut cultivated in Tunisia [64]. Interestingly, strain SZCCT0449 was unexpectedly isolated from the roots of a non-legume species, *Erigeron annuus*, in China [65]. This cluster also formed a separate monophyletic clade alongside three unidentified *Bradyrhizobium* species, all of which exhibited ANI values below the species delineation threshold of 96% when compared to 1AS2L^T^. This clade had a sister group consisting of three unidentified *Bradyrhizobium* species and four type strains of validly published species: *Bradyrhizobium symbiodeficiens* 85S1MB^T^, *Bradyrhizobium amphicarpaeae* 39S1MB^T^, *B. ottawaense* OO99^T^ and *B. shewense* ERR11^T^ (Fig. 4). The convergence of evidence from the distinct phylogenomic position, along with the results from previous analyses, strongly suggests that the Tunisian strains 1AS2L^T^, 1AS5L and 1AS20L represent a new species within the genus *Bradyrhizobium*.

**Fig. 5.**
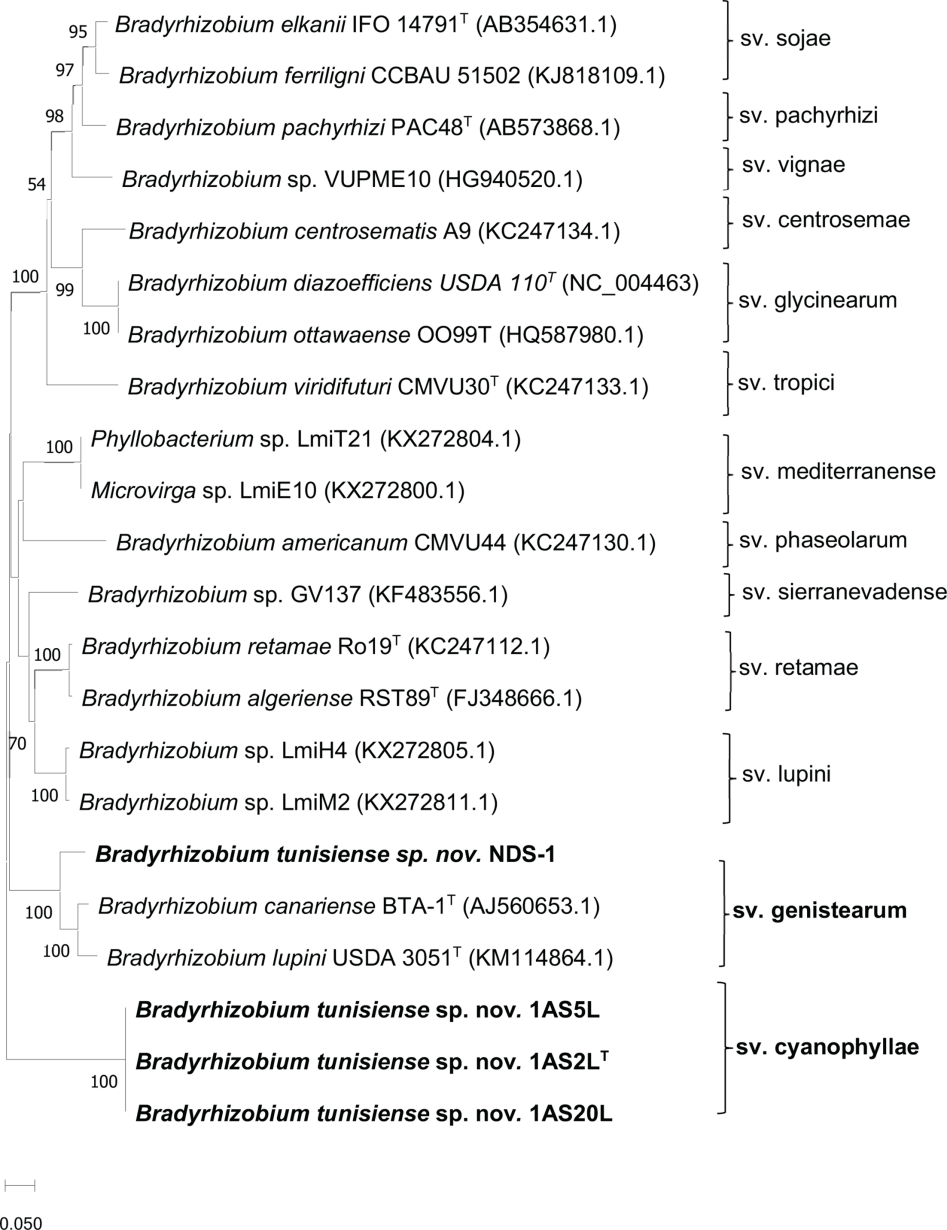
Phylogenetic trees of the *nodC* gene sequences (377 nucleotides) constructed using the ML method. The tree represents the symbiovars of *Bradyrhizobium* based on these sequences. Isolates of *B. tunisiense* sp. nov. are highlighted in bold. Bootstrap values ≥50 are indicated for each node (based on 1,000 replicates). The scale bar represents the number of substitutions per site.

Symbiotic analyses of the *nod*C gene sequence are routinely employed to assign symbiovars. Strains of *B. tunisiense* isolated from this study and strain NDS-1 were categorized into two distinct symbiotic groups (Fig. 5). The group comprises strains 1AS2L^T^, 1AS5L and 1AS20L, isolated from the root nodules of *A. saligna*. Based on the phylogeny of the *nodC* gene, which is involved in the nodulation process, these strains have been classified as belonging to the symbiovar cyanophyllae. Hsouna *et al*. [7] demonstrated that these strains are highly efficient in forming nodules and fixing nitrogen with several host plants, including *A. saligna*, *Acacia salicina*, *Leucaena leucocephala* and *Acacia tortilis*. However, their symbiotic efficiency varies among different plant species. For example, while they can nodulate *Phaseolus vulgaris*, they do not fix nitrogen with this plant. Additionally, they fail to nodulate *G. max* (soybean) and *Retama raetam*. The second group consists of strain NSD-1, which belongs to the symbiovar genistearum. This strain effectively forms nodules on peanut plants (*Arachis hypogaea*), indicating its potential use in legume cultivation and the improvement of soil fertility through biological nitrogen fixation [26].

Strain SZCCTO449, an endophyte isolated from the roots of the non-leguminous species, *E. annuus* in the Anhui region of China, could not be assigned to a given symbiovar since the *nod*C could not be obtained from the genome sequence. The reasons why no *nod*C gene was found are unknown. One reason could be that the genome sequence deposited in GenBank is incomplete. In fact, NCBI RefSeq staff recently suppressed the accession number GCA_018131185.1 indicating that ‘genome length too small’. It is also plausible but yet to be determined that stain SZCCTO449 lacks key symbiotic genes, such as the nodulation (*nod*) and nitrogen fixation (*nif*) genes, which are essential for root nodule formation and nitrogen fixation in leguminous plants. If true, this finding aligns with previous evidence that *Bradyrhizobium* species can also colonize non-leguminous species, such as rice [66,67], with significant implications for enhancing rice yield [68].

The categorization of *B. tunisiense* strains highlights the diversity of their symbiotic relationships and highlights their potential role in agriculture, particularly in enhancing nitrogen fixation across different plant species.

### Physiology, chemotaxonomy and cell morphology

Phenotypic characterization of the 1AS2L^T^, 1AS5L and 1AS20L strains was conducted in comparison with the type strains of * B. frederickii* CNPso 3426^T^, *B. shewense* ERR11^T^ and *B. ottawaense* OO99^T^, as these exhibited the highest dDDH and ANI values, although these values were below the species delineation cut-off (Table 3).

**Table 3.** Phenotypic tests performed using Biolog GEN III MicroPlates for the following strains at 28 °C: 1, 1AS20L; 2, 1AS2L; 3, 1AS5L; 4, *Bradyrhizobium ottawaense* strain OO99^T^; 5, *B. diazoefficiens* USDA 110^T^; 6, *B. frederickii* CNPSo 3426^T^; and 7, *B. shewense* strain ERR11^T^. The results are interpreted as follows: +, positive; −, negative; ±, weakly positive; nd, not determined

Biolog substrate	1	2	3	4	5	6	7
d-Fructose	−	−	−	−	−	+	−
d-Galactose	−	−	−	−	w	w	w
l-Fucose	+	+	+	−	w	+	+
d-Mannitol	−	−	−	−	−	w	+
d-Arabitol	+	+	+	+	−	+	+
d-Fructose-6-PO4	−	−	−	−	−	+	−
l-Aspartic acid	−	−	−	−	−	nd	+
l-Glutamic acid	−	−	−	−	w	nd	+
Citric acid	−	−	−	w	−	nd	+
*α*-Ketoglutaric acid	+	+	+	+	+	nd	−
Bromo-succinic acid	+	+	+	−	+	nd	+
Acetoacetic acid	+	+	+	−	−	nd	−
Propionic acid	−	−	−	−	+	nd	−
**Chemical sensitivity**	**1**	**2**	**3**	**4**	**5**	**6**	**7**
pH 5	−	−	−	+	+	+	+
Nalidixic acid	+	+	+	+	w	+	w
Potassium tellurite	+	+	+	−	±	nd	−
Aztreonam	+	+	+	w	−	nd	−
**Antibiotic resistance (µg ml^−1^)**	**1**	**2**	**3**	**4**	**5**	**6**	**7**
Erythromycin 15	+	+	+	+	−	−	nd
Ampicillin 10	+	+	+	nd	w	−	nd
Penicillin 10	+	+	+	−	nd	nd	nd

Several phenotypic and physiological features supported the clustering of the strains 1AS2L^T^, 1AS5L and 1AS20L. Colonies on YEM agar were circular, convex, white and measured less than 0.5 mm in diameter after 6 days at 28 °C. These strains were salt-sensitive and could not grow in more than 0.5% NaCl. They grew at pH 6–9 and at temperatures ranging from 16 to 37 °C, with optimal growth conditions at 28 °C and pH 7, consistent with many *Bradyrhizobium* species [68].

The carbon substrates utilized by all three strains of *B. tunisiense* sp. nov. were identical but differed from at least one of their closest related type strains, including the ability to assimilate l-fucose, d-arabitol, *α*-ketoglutaric acid, bromo-succinic acid and acetoacetic acid (Table 3). Conversely, these strains could not assimilate d-trehalose, gentiobiose, sucrose, d-turanose, d-raffinose, d-melibiose, *α*-d-glucose, d-mannose, d-fructose, d-galactose, d-mannitol, d-fructose-6-PO4, l-aspartic acid, l-glutamic acid, citric acid and propionic acid.

Additional distinguishing characteristics included positive enzymatic reactions for alkaline phosphatase and acid phosphatase, and a negative reaction for trypsin. The *B. tunisiense* sp. nov. strains were resistant to erythromycin (15 µg ml^−1^), ampicillin (10 µg ml^−1^) and penicillin (10 µg ml^−1^).

The major fatty acid in the type strain 1AS2L^T^ was summed feature 8 (C₁₈:₁ ω7c) at 76.58%, with a notable amount of C₁₆:₀ at 11.16% (Table S3). Additionally, small amounts of C₁₆:₁ ω5c (1.88%) and trace amounts of C₁₇:₁ ω8c, C₁₇:₀ and C₁₈:₁ ω5c were detected; these fatty acids were absent in *B. diazoefficiens* USDA 110ᵀ and *B. frederickii* CNPSo 3426ᵀ.

The cells of the type strain, 1AS2L^T^, of *B. tunisiense* sp. nov. are rod-shaped based on atomic force microscopy (Fig. S4).

Based on these polyphasic data (whole genomes, biochemical and phenotypic) and analyses, a new species, *B. tunisiense* sp. nov., is proposed.

## Description of *Bradyrhizobium tunisiense* sp. nov.

*Bradyrhizobium tunisiense* (tu.ni.si.en’se. N.L. neut. adj. tunisiense, belonging to or originating from Tunisia, the country from which the bacterium was isolated).

Cells are Gram-negative, aerobic, motile and non-spore-forming rods. Colonies on yeast extract mannitol agar (YMA) medium are less than 0.5 mm in diameter, circular, convex and whitish after incubation for 6 days at 28 °C. Optimal growth occurs at 28 °C, with growth observed at pH 6.0–9.0. Strains are salt-sensitive, tolerating NaCl concentrations up to 0.5% (w/v). The type strain, 1AS2Lᵀ, utilizes l-fucose, d-arabitol, *α*-ketoglutaric acid, bromo-succinic acid and acetoacetic acid but does not utilize d-trehalose, gentiobiose, sucrose, d-turanose, d-raffinose, d-melibiose, *α*-d-glucose, d-mannose, d-fructose, d-galactose, d-mannitol, d-fructose-6-phosphate, l-aspartic acid, l-glutamic acid, citric acid or propionic acid.

The type strain forms effective nodules on *A. saligna*, *A. salicina*, *L. leucocephala and A. tortilis* but forms ineffective nodules on *P. vulgaris*. No nodulation occurs with *G. max or R. raetam*. The predominant fatty acid is summed feature 8 (C₁₈:₁ ω7c). Strains 1AS20L and 1AS5L share similar phenotypic characteristics with the type strain and are also members of this species. Strains SZCCT0449 and NDS-1 in NCBI GenBank accession numbers GCA_018131185.1 and GCA_032918005.1, respectively, are putative members of *B. tunisiense* based on whole-genome data.

The genome of the type strain 1AS2Lᵀ is ~7,924,702 bp, with a G+C content of 63.87 mol%. Whole-genome sequences are available under the NCBI GenBank accession numbers JBHLEX01 (1AS2Lᵀ), JBHLEV01 (1AS20L) and JBHLEW01 (1AS5L). The GenBank accession numbers for the 16S rRNA, *atp*D, *rec*A, *gln*II, *gyr*B and *nod*C gene sequences of the type strain are OM392026, OM429371, OM429415, OM429425, OM429477 and OM429516, respectively.

The type strain, 1AS2Lᵀ (=LMG 33170ᵀ=DSM 114401ᵀ), was isolated from an effective nodule of *A. saligna* growing in soil from Borj Cedria, northeastern Tunisia.

## Supplementary material

10.1099/ijsem.0.006807 Uncited Supplementary Material 1.

## Abbreviations

ANI: average nucleotide identity
dDDH: digital DNA–DNA hybridization
GBDP: Genome blast Distance Phylogeny
GTDB: Genome Taxonomy Database
MAGs: metagenome-assembled genomes
ML: maximum-likelihood
TYGS: Type (Strain) Genome Server
UBCG2: up-to-date bacteria core genes pipeline version 2
YEM: yeast extract mannitol
YMA: yeast mannitol agar
