# Supplementary material for: Bradyrhizobium tunisiense sp. nov., a novel rhizobial species isolated from Acacia saligna nodules
Source: Int J Syst Evol Microbiol. 2025 Jun 19;75(6):006807. doi: 10.1099/ijsem.0.006807 (PMC12179390; doi:10.1099/ijsem.0.006807)
Supplement: Uncited Supplementary Material 1. [file ijsem-75-06807-s001.pdf]

Supplementary materials - Tables and Figures.

Bradyrhizobium tunisiense **sp. nov., a novel rhizobial species isolated from** ***Acacia saligna*** **nodules**

Jihed Hsouna[1], Houda Zouagui[2], Takwa Gritli[1], Houda Ilahi[1], Jia-Cheng Han[3], Muhammad Sulman[4], Walid Ellouze[4], Xiao Xia Zhang[3], Maroua Mansouri[1], Mustapha Missbah El Idrissi[2], Soufiane Alami[2], Pierre Emmanuel Courty[5], Daniel Wipf[5], Abdelkader Bekki[6], James T. Tambong[7]* and Bacem Mnasri[1]*

International Journal of Systematics and Evolutionary Microbiology.

Table S1. OrthoANI and dDDH values estimated between 1AS2L^{T} and 225 *Bradyrhizobium* metagenome-assembled genomes (MAGs)

Accession	Organism Name	Organism Infraspecific Names Isolate	OrthoANI (%)	dDDH (%)
GCA_002341725.1	Bradyrhizobium sp.	UBA2491	80.384	23.70
GCA_013361965.1	Bradyrhizobium sp.	Bin4.9.1.1	89.218	38.40
GCA_013695565.1	Bradyrhizobium sp.	MGR_bin250	79.0237	22.80
GCA_013822885.1	Bradyrhizobium sp.	PB1.002	78.8771	23.00
GCA_016213215.1	Bradyrhizobium sp.	NC_groundwater_1604_Pr3_B-0.1um_63_21	79.8903	23.10
GCA_016219575.1	Bradyrhizobium sp.	NC_groundwater_1901_Pr3_S2p5_64_30	79.8184	23.10
GCA_016462955.1	Bradyrhizobium sp.	SupBloom_Metag_066	87.2094	33.90
GCA_017881085.1	Bradyrhizobium sp.	Chersky.37_22	79.2045	22.60
GCA_019242285.1	Bradyrhizobium sp.	CP_BM_RX_45	79.2242	22.90
GCA_019243075.1	Bradyrhizobium sp.	CP_BM_RX_19	79.0025	22.20
GCA_019244325.1	Bradyrhizobium sp.	CP_BM_ER_R8_28	78.9016	22.90
GCA_019510065.1	Bradyrhizobium sp.	SM_64_9	84.2783	32.70
GCA_020252445.1	Bradyrhizobium sp.	M19BS1SP1A50MG	80.2795	23.90
GCA_020252455.1	Bradyrhizobium sp.	M20BS1SP1A50MG	80.4298	23.90
GCA_020350345.1	Bradyrhizobium sp.	bin51	86.6071	32.00
GCA_021153675.1	Bradyrhizobium sp.	b94_HoplandSoilSeptJan	84.6334	30.60
GCA_021732175.1	Bradyrhizobium sp.	D3_Bin_4	79.3951	23.10
GCA_022843365.1	Bradyrhizobium sp.	Bin_71_2	78.9352	22.10
GCA_022844465.1	Bradyrhizobium sp.	Bin_23_1	79.1123	22.40
GCA_024231615.1	Bradyrhizobium sp.	Gpa_14	79.3834	22.90
GCA_028868675.1	Bradyrhizobium sp.	REEB386	83.6276	27.00
GCA_030626485.1	Bradyrhizobium sp.	CSMAG_85	80.0775	23.80
GCA_030646015.1	Bradyrhizobium sp.	E19_concoct.bin.29	79.2515	22.60
GCA_030653055.1	Bradyrhizobium sp.	E6_concoct.bin.93_sub	78.9578	23.40
GCA_030654605.1	Bradyrhizobium sp.	E10_concoct.bin.67	79.392	22.60
GCA_030679815.1	Bradyrhizobium sp.	C13_concoct.bin.107_sub	79.6573	24.20
GCA_030681415.1	Bradyrhizobium sp.	C12_metabat.bin.4	78.9968	22.60
GCA_030693485.1	Bradyrhizobium sp.	C18_metabat.bin.89	79.1467	22.50
GCA_030697065.1	Bradyrhizobium sp.	C5_concoct.bin.166	79.1096	22.50
GCA_032470795.1	Bradyrhizobium sp.	N4_207_000G1_dasN4_207_000G1_concoct_42	78.7952	23.80
GCA_032474435.1	Bradyrhizobium sp.	N4_160_000G1_dasN4_160_000G1_concoct_37	78.7216	22.70
GCA_032474735.1	Bradyrhizobium sp.	N4_159_000G1_dasN4_159_000G1_concoct_32	78.8347	22.80
GCA_032475555.1	Bradyrhizobium sp.	N4_154_000G1_dasN4_154_000G1_concoct_33	78.6116	23.40
GCA_032475895.1	Bradyrhizobium sp.	N4_152_006G1_dasN4_152_006G1_concoct_22	78.6997	23.80
GCA_032476235.1	Bradyrhizobium sp.	N4_151_000G1_dasN4_151_000G1_concoct_20	78.6722	22.90
GCA_032485715.1	Bradyrhizobium sp.	N4_213_000G1_dasN4_213_000G1_concoct_13	78.8122	23.10
GCA_032486215.1	Bradyrhizobium sp.	N4_211_016G1_dasN4_211_016G1_concoct_14	78.7942	23.20
GCA_032486755.1	Bradyrhizobium sp.	N4_131_000G1_dasN4_131_000G1_concoct_0	78.8904	22.80
GCA_032487015.1	Bradyrhizobium sp.	N4_129_016G1_dasN4_129_016G1_concoct_10	78.8286	23.50
GCA_032487415.1	Bradyrhizobium sp.	N4_128_000G1_dasN4_128_000G1_concoct_5	78.6579	22.70
GCA_032563275.1	Bradyrhizobium sp.	N4_126_000G1_dasN4_126_000G1_concoct_35	78.766	22.70
GCA_032563695.1	Bradyrhizobium sp.	N4_125_000G1_dasN4_125_000G1_concoct_22	78.6299	23.20
GCA_032564235.1	Bradyrhizobium sp.	N4_124_000G1_dasN4_124_000G1_concoct_19	78.7176	22.80
GCA_032565015.1	Bradyrhizobium sp.	N4_115_000G1_dasN4_115_000G1_concoct_34	78.7684	22.80
GCA_032565875.1	Bradyrhizobium sp.	N4_106_000G1_dasN4_106_000G1_maxbin2.max	78.8028	22.70
GCA_032566575.1	Bradyrhizobium sp.	N4_105_000G1_dasN4_105_000G1_maxbin2.max	78.6265	22.70
GCA_032567255.1	Bradyrhizobium sp.	N4_104_000G1_dasN4_104_000G1_concoct_71	78.778	23.30
GCA_032568155.1	Bradyrhizobium sp.	N4_101_000G1_dasN4_101_000G1_maxbin2.max	78.7513	23.60
GCA_032568475.1	Bradyrhizobium sp.	N4_100_000G1_dasN4_100_000G1_abawaca.13	78.6822	22.70
GCA_032568985.1	Bradyrhizobium sp.	N4_097_000G1_dasN4_097_000G1_maxbin2.max	78.7597	22.70
GCA_032569235.1	Bradyrhizobium sp.	N4_095_016G1_dasN4_095_016G1_concoct_6	78.6086	24.30
GCA_032570695.1	Bradyrhizobium sp.	N4_089_000G1_dasN4_089_000G1_concoct_15	78.8566	22.80
GCA_032571055.1	Bradyrhizobium sp.	N4_087_009G1_dasN4_087_009G1_concoct_33	78.7178	23.50
GCA_033795385.1	Bradyrhizobium sp.	MAG10_BQ13	88.1201	35.50
GCA_034669345.1	Bradyrhizobium sp.	BP_669	79.1398	22.60
GCA_035277285.1	Bradyrhizobium sp.	SMAG_U8898	79.328	22.40
GCA_035278345.1	Bradyrhizobium sp.	SMAG_U8854	79.0423	22.80
GCA_035293585.1	Bradyrhizobium sp.	SMAG_U9913	79.4045	23.10
GCA_035309285.1	Bradyrhizobium sp.	SMAG_U9723	78.9162	22.50
GCA_035325405.1	Bradyrhizobium sp.	AMR_MDS_0061	79.4889	23.00
GCA_035484855.1	Bradyrhizobium sp.	SMAG_U12687	78.7625	22.40
GCA_035487555.1	Bradyrhizobium sp.	SMAG_U12620	79.4495	22.80
GCA_035498775.1	Bradyrhizobium sp.	SMAG_U15188	78.7502	22.60

GCA_035544035.1	Bradyrhizobium sp.	SMAG_U14606	78.6347	22.80
GCA_035566995.1	Bradyrhizobium sp.	SMAG_U12911	78.799	22.50
GCA_035568535.1	Bradyrhizobium sp.	SMAG_U12859	79.5216	22.90
GCA_035575905.1	Bradyrhizobium sp.	SMAG_U13245	79.719	22.90
GCA_035575945.1	Bradyrhizobium sp.	SMAG_U13239	87.1968	33.60
GCA_035602495.1	Bradyrhizobium sp.	SMAG_U11486	78.8622	22.50
GCA_035652015.1	Bradyrhizobium sp.	SMAG_U10009	79.0641	22.60
GCA_035935975.1	Bradyrhizobium sp.	SMAG_U6282	87.4058	33.80
GCA_035995625.1	Bradyrhizobium sp.	SMAG_U4317	78.6815	22.30
GCA_036258215.1	Bradyrhizobium sp.	SMAG_U6767	79.1249	23.00
GCA_036266415.1	Bradyrhizobium sp.	SMAG_U7006	78.7191	22.10
GCA_036383375.1	Bradyrhizobium sp.	SMAG_U3152	80.1239	24.00
GCA_036390315.1	Bradyrhizobium sp.	SMAG_U2697	86.1461	31.70
GCA_036406295.1	Bradyrhizobium sp.	SMAG_U3626	79.4525	22.90
GCA_036497145.1	Bradyrhizobium sp.	SMAG_U5263	78.6522	22.30
GCA_036511315.1	Bradyrhizobium sp.	SMAG_U7697	78.6997	22.50
GCA_036554215.1	Bradyrhizobium sp.	SMAG_U4030	79.2885	22.70
GCA_036565985.1	Bradyrhizobium sp.	SMAG_U7431	79.2015	23.20
GCA_036721875.1	Bradyrhizobium sp.	CTOTU4207	78.8349	23.30
GCA_036735945.1	Bradyrhizobium sp.	CTOTU2283	79.4889	23.00
GCA_039649085.1	Bradyrhizobium sp.	Holy_361	80.3017	23.90
GCA_039922755.1	Bradyrhizobium sp.	cluster2_bin.195	78.6203	22.20
GCA_902482845.1	uncultured Bradyrhizobium sp.	MGYG-HGUT-03137	78.7	22.70
GCA_902488645.1	uncultured Bradyrhizobium sp.	MGYG-HGUT-03733	80.2576	23.80
GCA_902826195.1	uncultured Bradyrhizobium sp.	Bradyrhizobium sp. RBC079	79.1055	22.90
GCA_936920605.1	uncultured Bradyrhizobium sp.	SRR6145093_bin.62_CONCOCT_v1.1_MAG	80.1226	23.60
GCA_936928535.1	uncultured Bradyrhizobium sp.	ERR2144899_bin.10_CONCOCT_v1.1_MAG	85.6491	30.40
GCA_936943645.1	uncultured Bradyrhizobium sp.	ERR2144886_bin.2_CONCOCT_v1.1_MAG	88.8397	36.80
GCA_937872855.1	uncultured Bradyrhizobium sp.	SRR6158312_bin.26_CONCOCT_v1.1_MAG	80.3615	23.80
GCA_946404825.1	Bradyrhizobium diazoefficiens	DRR089564_bin.1_metawrap_v1.3.0_MAG	87.71	34.90
GCA_946415105.1	Bradyrhizobium elkanii	SRR9694907_bin.1_metawrap_v1.3.0_MAG	80.3461	24.10
GCA_947040575.1	uncultured Bradyrhizobium sp.	ERR3047202_bin.2_metaWRAP_v1.3_MAG	87.4331	34.10
GCA_947648125.1	uncultured Bradyrhizobium sp.	SRR15633984_bin.32_metawrap_v1.3_MAG	80.1961	23.70
GCA_963512315.1	uncultured Bradyrhizobium sp.	SRR17798732_bin.7_MetaWRAP_v1.3_MAG	80.2247	23.60
GCA_963543985.1	Bradyrhizobium viridifuturi	SRR17738114_bin.7_MetaWRAP_v1.3_MAG	80.434	23.80
GCA_004799405.1	Bradyrhizobium sp.	LY.8	78.439	21.90
GCA_004799445.1	Bradyrhizobium sp.	LY.54	78.3386	21.90
GCA_018971675.1	Bradyrhizobium sp.	REEB70	78.2623	21.70
GCA_019232395.1	Bradyrhizobium sp.	CP_BE_RX_R3_8	78.241	22.60
GCA_019244115.1	Bradyrhizobium sp.	CP_BM_ER_R9_4	78.4125	22.60
GCA_019247825.1	Bradyrhizobium sp.	CP_BM_RX_R9_26	78.3894	22.50
GCA_025936355.1	Bradyrhizobium sp.	GZL_HYHR_2_bin.116	77.5434	21.80
GCA_026125985.1	Bradyrhizobium sp.	TGS_PRO17	78.4679	22.50
GCA_026225025.1	Bradyrhizobium sp.	NGEE_A_bin38	77.6336	22.30
GCA_027307225.1	Bradyrhizobium sp.	clean1649	77.766	22.00
GCA_028290305.1	Bradyrhizobium sp.	RYN_501	77.5332	21.60
GCA_028869075.1	Bradyrhizobium sp.	REEB367	78.4408	22.10
GCA_028870055.1	Bradyrhizobium sp.	REEB301	77.4617	21.70
GCA_028870895.1	Bradyrhizobium sp.	REEB259	77.7742	21.60
GCA_028872135.1	Bradyrhizobium sp.	REEB177	78.0329	21.50
GCA_030150345.1	Bradyrhizobium sp.	SHSU.bins.399	78.4058	22.80
GCA_030655565.1	Bradyrhizobium sp.	E23_concoct.bin.33_sub	78.2543	22.30
GCA_030681365.1	Bradyrhizobium sp.	C15_maxbin.089_sub	78.2917	21.90
GCA_031291485.1	Bradyrhizobium sp.	MAG_BA244	77.7512	21.70
GCA_032564835.1	Bradyrhizobium sp.	N4_116_000G1_dasN4	78.4881	24.10
GCA_032565655.1	Bradyrhizobium sp.	N4_107_000G1_da	78.1556	24.60
GCA_032570165.1	Bradyrhizobium sp.	N4_094_000G1_dasN	78.3259	24.90
GCA_034669205.1	Bradyrhizobium sp.	BP_675	78.0439	22.00
GCA_034669225.1	Bradyrhizobium sp.	BP_673	77.9337	22.50
GCA_034669245.1	Bradyrhizobium sp.	BP_672	78.0523	22.10
GCA_034669265.1	Bradyrhizobium sp.	BP_674	78.0733	22.10
GCA_034669285.1	Bradyrhizobium sp.	BP_671	78.0278	22.30
GCA_034669305.1	Bradyrhizobium sp.	BP_670	77.516	21.30
GCA_034669365.1	Bradyrhizobium sp.	BP_667	77.6957	21.40
GCA_034669385.1	Bradyrhizobium sp.	BP_666	77.897	22.00
GCA_034669405.1	Bradyrhizobium sp.	BP_668	77.5076	21.70

GCA_034669445.1	Bradyrhizobium sp.	BP_664	77.6235	21.60
GCA_034669465.1	Bradyrhizobium sp.	BP_663	77.5482	21.90
GCA_035258325.1	Bradyrhizobium sp.	SMAG_U8377	78.4765	22.80
GCA_035308285.1	Bradyrhizobium sp.	SMAG_U9766	78.128	21.80
GCA_035317645.1	Bradyrhizobium sp.	SMAG_U12472	78.1689	22.70
GCA_035317665.1	Bradyrhizobium sp.	SMAG_U12471	78.0438	22.90
GCA_035317685.1	Bradyrhizobium sp.	SMAG_U12471	77.9011	22.60
GCA_035317745.1	Bradyrhizobium sp.	SMAG_U12468	77.8163	22.40
GCA_035317835.1	Bradyrhizobium sp.	SMAG_U12461	78.0668	22.60
GCA_035489795.1	Bradyrhizobium sp.	SMAG_U12551	77.6745	22.60
GCA_035490015.1	Bradyrhizobium sp.	SMAG_U12517	78.0908	22.10
GCA_035490095.1	Bradyrhizobium sp.	SMAG_U12504	77.8777	21.90
GCA_035490115.1	Bradyrhizobium sp.	SMAG_U12496	77.4752	22.20
GCA_035535695.1	Bradyrhizobium sp.	SMAG_U13857	77.9156	22.00
GCA_035538775.1	Bradyrhizobium sp.	SMAG_U13703	77.3942	22.00
GCA_035547995.1	Bradyrhizobium sp.	SMAG_U6077	78.3282	22.50
GCA_035555355.1	Bradyrhizobium sp.	SMAG_U13496	78.5643	22.90
GCA_035558295.1	Bradyrhizobium sp.	SMAG_U13385	77.9362	22.00
GCA_035566795.1	Bradyrhizobium sp.	SMAG_U12929	78.3786	21.90
GCA_035570375.1	Bradyrhizobium sp.	SMAG_U12807	77.5916	21.80
GCA_035601375.1	Bradyrhizobium sp.	SMAG_U11527	77.8492	21.80
GCA_035601555.1	Bradyrhizobium sp.	SMAG_U11520	78.0642	21.60
GCA_035603875.1	Bradyrhizobium sp.	SMAG_U11400	78.2162	22.50
GCA_035604015.1	Bradyrhizobium sp.	SMAG_U11395	78.1245	22.30
GCA_035629815.1	Bradyrhizobium sp.	SMAG_U11948	77.7994	22.30
GCA_035636175.1	Bradyrhizobium sp.	SMAG_U11623	77.7994	22.10
GCA_035639035.1	Bradyrhizobium sp.	SMAG_U11074	78.1566	22.20
GCA_035643075.1	Bradyrhizobium sp.	SMAG_U10874	77.7475	22.40
GCA_035644295.1	Bradyrhizobium sp.	SMAG_U10811	77.836	22.10
GCA_035652035.1	Bradyrhizobium sp.	SMAG_U10008	77.7466	21.80
GCA_035693465.1	Bradyrhizobium sp.	SMAG_U1033	77.8982	21.80
GCA_035695385.1	Bradyrhizobium sp.	SMAG_U973	77.6514	21.70
GCA_035701725.1	Bradyrhizobium sp.	SMAG_U492	77.847	22.00
GCA_035714265.1	Bradyrhizobium sp.	SMAG_U3495	78.1201	22.30
GCA_035714325.1	Bradyrhizobium sp.	SMAG_U3523	77.9918	22.30
GCA_035719885.1	Bradyrhizobium sp.	SMAG_U6	77.9585	22.30
GCA_035949555.1	Bradyrhizobium sp.	SMAG_U6579	78.5354	22.00
GCA_035995945.1	Bradyrhizobium sp.	SMAG_U4309	78.3039	22.30
GCA_035998015.1	Bradyrhizobium sp.	SMAG_U4253	77.9888	22.00
GCA_036009065.1	Bradyrhizobium sp.	SMAG_U4376	78.3624	22.20
GCA_036273255.1	Bradyrhizobium sp.	SMAG_U1448	78.1695	21.60
GCA_036393755.1	Bradyrhizobium sp.	SMAG_U2816	77.5646	22.00
GCA_036394735.1	Bradyrhizobium sp.	SMAG_U2762	77.9854	21.90
GCA_036401355.1	Bradyrhizobium sp.	SMAG_U5258	78.2851	23.30
GCA_036497205.1	Bradyrhizobium sp.	SMAG_U4891	77.9967	21.80
GCA_036501115.1	Bradyrhizobium sp.	SMAG_U4853	78.0826	21.70
GCA_036501915.1	Bradyrhizobium sp.	SMAG_U4814	78.1091	22.10
GCA_036502725.1	Bradyrhizobium sp.	SMAG_U4653	77.9642	22.10
GCA_036506295.1	Bradyrhizobium sp.	SMAG_U7637	78.2743	21.80
GCA_036512795.1	Bradyrhizobium sp.	SMAG_U7594	77.7677	23.00
GCA_036513695.1	Bradyrhizobium sp.	SMAG_U7594	77.7635	21.80
GCA_036768605.1	Bradyrhizobium sp.	CTOTU41419	78.3919	22.40
GCA_037201505.1	Bradyrhizobium sp.	S6_metab_bin.109	77.8919	21.80
GCA_039649215.1	Bradyrhizobium sp.	Holy_362	78.1271	22.50
GCA_039922595.1	Bradyrhizobium sp.	cluster2_bin.20	78.3601	22.10
GCA_039932655.1	Bradyrhizobium sp.	cluster3_bin.201	78.0262	22.20
GCA_039935075.1	Bradyrhizobium sp.	cluster2_bin.9	78.3658	22.10
GCA_039935515.1	Bradyrhizobium sp.	cluster2_bin.69	78.5542	22.20
GCA_040004195.1	Bradyrhizobium sp.	cluster4_bin.212	77.7457	22.10
GCA_002280915.1	Bradyrhizobium sp.	35-63-5	72.8185	20.30
GCA_004799435.1	Bradyrhizobium sp.	LY.7	70.6567	19.00
GCA_019242075.1	Bradyrhizobium sp.	CP_BM_RX_56	77.1032	21.70
GCA_019509805.1	Bradyrhizobium sp.	VL_62_9	76.8847	21.90
GCA_025936395.1	Bradyrhizobium sp.	GZL_HYHR_2_bin.111	76.637	21.00
GCA_025960445.1	Bradyrhizobium sp.	GZL_FP_2_bin.119	76.7196	21.00
GCA_027430895.1	Bradyrhizobium sp.	clean6046	77.0886	21.70

GCA_027430915.1	Bradyrhizobium sp.	clean6045	77.1656	21.80
GCA_027430935.1	Bradyrhizobium sp.	clean6043	77.1425	21.70
GCA_027430955.1	Bradyrhizobium sp.	clean6044	76.8397	21.50
GCA_027430975.1	Bradyrhizobium sp.	clean6042	77.1534	21.70
GCA_027451485.1	Bradyrhizobium sp.	clean379	77.1704	21.80
GCA_028290165.1	Bradyrhizobium sp.	RYN_508	77.2502	21.30
GCA_028290585.1	Bradyrhizobium sp.	RYN_486	77.1633	21.80
GCA_028865105.1	Bradyrhizobium sp.	REEB172	77.1583	21.90
GCA_028866295.1	Bradyrhizobium sp.	REEB116	77.1975	21.70
GCA_028869655.1	Bradyrhizobium sp.	REEB329	76.913	21.00
GCA_030151605.1	Bradyrhizobium sp.	SHSU.bins.164	76.7424	21.40
GCA_031367195.1	Bradyrhizobium sp.	OTE_38_metabat_380	70.5534	18.00
GCA_034669425.1	Bradyrhizobium sp.	BP_665	77.1599	21.40
GCA_035264965.1	Bradyrhizobium sp.	SMAG_U9297	76.7165	21.30
GCA_035273305.1	Bradyrhizobium sp.	SMAG_U9017	76.3427	20.70
GCA_035295025.1	Bradyrhizobium sp.	SMAG_U9839	76.7897	21.20
GCA_035482015.1	Bradyrhizobium sp.	SMAG_U12758	76.8039	21.70
GCA_035483915.1	Bradyrhizobium sp.	SMAG_U12709	76.9789	21.30
GCA_035524175.1	Bradyrhizobium sp.	SMAG_U14787	77.2158	21.90
GCA_035567775.1	Bradyrhizobium sp.	SMAG_U12899	76.7681	21.20
GCA_035688545.1	Bradyrhizobium sp.	SMAG_U1210	76.035	20.90
GCA_036008505.1	Bradyrhizobium sp.	SMAG_U4405	77.3354	21.70
GCA_036263175.1	Bradyrhizobium sp.	SMAG_U6859	76.5498	20.40
GCA_036385675.1	Bradyrhizobium sp.	SMAG_U3030	77.3768	21.90
GCA_036394295.1	Bradyrhizobium sp.	SMAG_U2786	77.0559	21.60
GCA_040392305.1	Bradyrhizobium sp.	T2.bin.282	75.5573	20.60
GCA_040511085.1	Bradyrhizobium sp.	T2R5UO_autometa_bin_0062	77.2539	21.90
GCA_943372355.1	Bradyrhizobium ivorense	JJ-MIC7-10-1-m1-bin347	74.8017	21.00
GCA_943372615.1	Bradyrhizobium ivorense	JJ-ME9A6-316-9-m2-bin108	76.3673	21.20
GCA_943372745.1	Bradyrhizobium ivorense	JJ-MO7B3-363-8-m2-bin124	76.5447	20.80

Supplementary Table S2. One hundred and seventeen bacterial gene markers used in GTDB-TK pipeline to infer a phylogenomic tree for the genus *Bradyrhizobium* .

Marker ID	Description
PF00380.20	Ribosomal protein S9/S16
PF00410.20	Ribosomal protein S8
PF00466.21	Ribosomal protein L10
PF01025.20	GrpE
PF02576.18	RimP N-terminal domain
PF03726.15	Polyribonucleotide nucleotidyltransferase, RNA binding domain
TIGR00006	TIGR00006: 16S rRNA (cytosine(1402)-N(4))-methyltransferase
TIGR00019	prfA: peptide chain release factor 1
TIGR00020	prfB: peptide chain release factor 2
TIGR00029	S20: ribosomal protein bS20
TIGR00043	TIGR00043: rRNA maturation RNase YbeY
TIGR00054	TIGR00054: RIP metalloprotease RseP
TIGR00059	L17: ribosomal protein bL17
TIGR00061	L21: ribosomal protein bL21
TIGR00064	ftsY: signal recognition particle-docking protein FtsY
TIGR00065	ftsZ: cell division protein FtsZ
TIGR00082	rbfA: ribosome-binding factor A
TIGR00083	ribF: riboflavin biosynthesis protein RibF
TIGR00084	ruvA: Holliday junction DNA helicase RuvA
TIGR00086	smpB: SsrA-binding protein
TIGR00088	trmD: tRNA (guanine(37)-N(1))-methyltransferase
TIGR00090	rsfS_iojap_ybeB: ribosome silencing factor
TIGR00092	TIGR00092: GTP-binding protein YchF
TIGR00095	TIGR00095: 16S rRNA (guanine(966)-N(2))-methyltransferase RsmD
TIGR00115	tig: trigger factor
TIGR00116	tsf: translation elongation factor Ts
TIGR00138	rsmG_gidB: 16S rRNA (guanine(527)-N(7))-methyltransferase RsmG
TIGR00158	L9: ribosomal protein bL9
TIGR00166	S6: ribosomal protein bS6
TIGR00168	infC: translation initiation factor IF-3
TIGR00186	rRNA_methyl_3: RNA methyltransferase, TrmH family, group 3
TIGR00194	uvrC: excinuclease ABC subunit C
TIGR00250	RNAse_H_YqgF: putative transcription antitermination factor YqgF
TIGR00337	PyrG: CTP synthase
TIGR00344	alaS: alanine--tRNA ligase
TIGR00362	DnaA: chromosomal replication initiator protein DnaA
TIGR00392	ileS: isoleucine--tRNA ligase
TIGR00396	leuS_bact: leucine--tRNA ligase
TIGR00398	metG: methionine--tRNA ligase
TIGR00414	serS: serine--tRNA ligase
TIGR00416	sms: DNA repair protein RadA
TIGR00420	trmU: tRNA (5-methylaminomethyl-2-thiouridylate)-methyltransferase
TIGR00431	TruB: tRNA pseudouridine(55) synthase
TIGR00435	cysS: cysteine--tRNA ligase
TIGR00436	era: GTP-binding protein Era
TIGR00442	hisS: histidine--tRNA ligase
TIGR00445	mraY: phospho-N-acetylmuramoyl-pentapeptide-transferase
TIGR00456	argS: arginine--tRNA ligase
TIGR00459	aspS_bact: aspartate--tRNA ligase
TIGR00460	fmt: methionyl-tRNA formyltransferase
TIGR00468	pheS: phenylalanine--tRNA ligase, alpha subunit
TIGR00472	pheT_bact: phenylalanine--tRNA ligase, beta subunit
TIGR00487	IF-2: translation initiation factor IF-2
TIGR00496	frr: ribosome recycling factor
TIGR00539	hemN_rel: putative oxygen-independent coproporphyrinogen III oxidase
TIGR00593	pola: DNA polymerase I
TIGR00615	recR: recombination protein RecR

TIGR00631	uvrb: excinuclease ABC subunit B
TIGR00634	recN: DNA repair protein RecN
TIGR00635	ruvB: Holliday junction DNA helicase RuvB
TIGR00643	recG: ATP-dependent DNA helicase RecG
TIGR00663	dnan: DNA polymerase III, beta subunit
TIGR00717	rpsA: ribosomal protein bS1
TIGR00755	ksgA: ribosomal RNA small subunit methyltransferase A
TIGR00810	secG: preprotein translocase, SecG subunit
TIGR00922	nusG: transcription termination/antitermination factor NusG
TIGR00959	ffh: signal recognition particle protein
TIGR00963	secA: preprotein translocase, SecA subunit
TIGR00964	secE_bact: preprotein translocase, SecE subunit
TIGR00967	3a0501s007: preprotein translocase, SecY subunit
TIGR01009	rpsC_bact: ribosomal protein uS3
TIGR01011	rpsB_bact: ribosomal protein uS2
TIGR01017	rpsD_bact: ribosomal protein uS4
TIGR01021	rpsE_bact: ribosomal protein uS5
TIGR01029	rpsG_bact: ribosomal protein uS7
TIGR01032	rplT_bact: ribosomal protein bL20
TIGR01039	atpD: ATP synthase F1, beta subunit
TIGR01044	rplV_bact: ribosomal protein uL22
TIGR01059	gyrB: DNA gyrase, B subunit
TIGR01063	gyrA: DNA gyrase, A subunit
TIGR01066	rplM_bact: ribosomal protein uL13
TIGR01071	rplO_bact: ribosomal protein uL15
TIGR01079	rplX_bact: ribosomal protein uL24
TIGR01082	murC: UDP-N-acetylmuramate--L-alanine ligase
TIGR01087	murD: UDP-N-acetylmuramoylalanine--D-glutamate ligase
TIGR01128	holA: DNA polymerase III, delta subunit
TIGR01146	ATPsyn_F1gamma: ATP synthase F1, gamma subunit
TIGR01164	rplP_bact: ribosomal protein uL16
TIGR01169	rplA_bact: ribosomal protein uL1
TIGR01171	rplB_bact: ribosomal protein uL2
TIGR01302	IMP_dehydrog: inosine-5'-monophosphate dehydrogenase
TIGR01391	dnaG: DNA primase
TIGR01393	lepA: elongation factor 4
TIGR01394	TypA_BipA: GTP-binding protein TypA/BipA
TIGR01510	coaD_prev_kdtB: pantetheine-phosphate adenylyltransferase
TIGR01632	L11_bact: ribosomal protein uL11
TIGR01951	nusB: transcription antitermination factor NusB
TIGR01953	NusA: transcription termination factor NusA
TIGR02012	tigrfam_recA: protein RecA
TIGR02013	rpoB: DNA-directed RNA polymerase, beta subunit
TIGR02027	rpoA: DNA-directed RNA polymerase, alpha subunit
TIGR02075	pyrH_bact: UMP kinase
TIGR02191	RNaseIII: ribonuclease III
TIGR02273	16S_RimM: 16S rRNA processing protein RimM
TIGR02350	prok_dnaK: chaperone protein DnaK
TIGR02386	rpoC_TIGR: DNA-directed RNA polymerase, beta' subunit
TIGR02397	dnaX_nterm: DNA polymerase III, subunit gamma and tau
TIGR02432	lysidine_TilS_N: tRNA(Ile)-lysidine synthetase
TIGR02729	Obg_CgtA: Obg family GTPase CgtA
TIGR03263	guanyl_kin: guanylate kinase
TIGR03594	GTPase_EngA: ribosome-associated GTPase EngA
TIGR03625	L3_bact: 50S ribosomal protein uL3
TIGR03632	uS11_bact: ribosomal protein uS11
TIGR03654	L6_bact: ribosomal protein uL6
TIGR03723	T6A_TsaD_YgjD: tRNA threonylcarbamoyl adenosine modification protein TsaD
TIGR03725	T6A_YeaZ: tRNA threonylcarbamoyl adenosine modification protein YeaZ
TIGR03953	rplD_bact: 50S ribosomal protein uL4

Table S3. Comparative analysis of the fatty acid composition (%) for the following strains: 1, 1AS2L^T; 2, *Bradyrhizobium ottawaense* OO99^T; 3, *Bradyrhizobium diazoefficiens* USDA 110^T; and 4, *Bradyrhizobium frederickii* CNPSo 3426^T

Fatty Acid	1	2*	3*	4**
$C_{16:1\ \omega}5c$	**1.88**	**6.3**	-	-
$C_{16:0}$	11.16	7.9	14.1	15.39
$C_{17:1\ \omega}8c$	tr	tr	-	-
**$C_{17:1\omega}6c$**	**tr**	**-**	**-**	**-**
$C_{17:00}$	tr	tr	-	-
$C_{18:1\omega}5c$	tr	-	-	-
$C_{18:0}$	tr	tr	-	-
**Summed Feature 3 ($C_{16:1\ \omega7c}$/$C_{16:1\ \omega6c}$)**	**1.77**	**1.6**	**-**	**-**
Summed Feature 8 ($C_{18:1\ \omega7c}$)	76.58	82.3	85.9	84.61

Tr: traces (Values below 1%)

*Data from Yu et al . (2014) [64]

** Data from Urquiaga et al. 2019 [58]

Figure S1

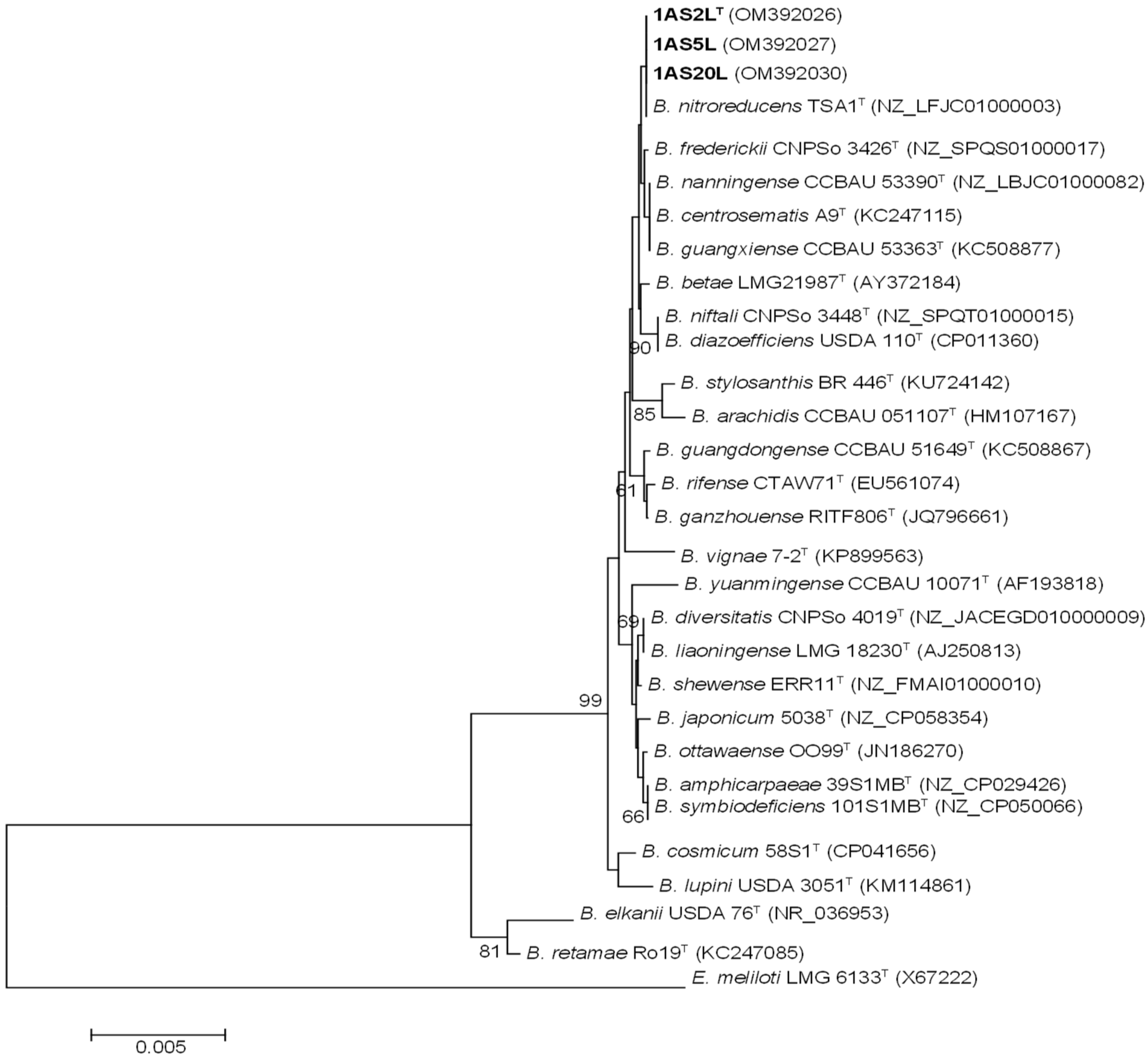

Figure S1. Maximum Likelihood-based phylogenetic analysis of *rrs* sequences (1215 nucleotides). The three novel strains are highlighted in bold. Bootstrap values ≥50 are indicated for each node based on 1000 replicates. Each species name is followed by the strain designation and the NCBI accession number of the sequence used. The scale bar represents the number of substitutions per site.

Figure S2

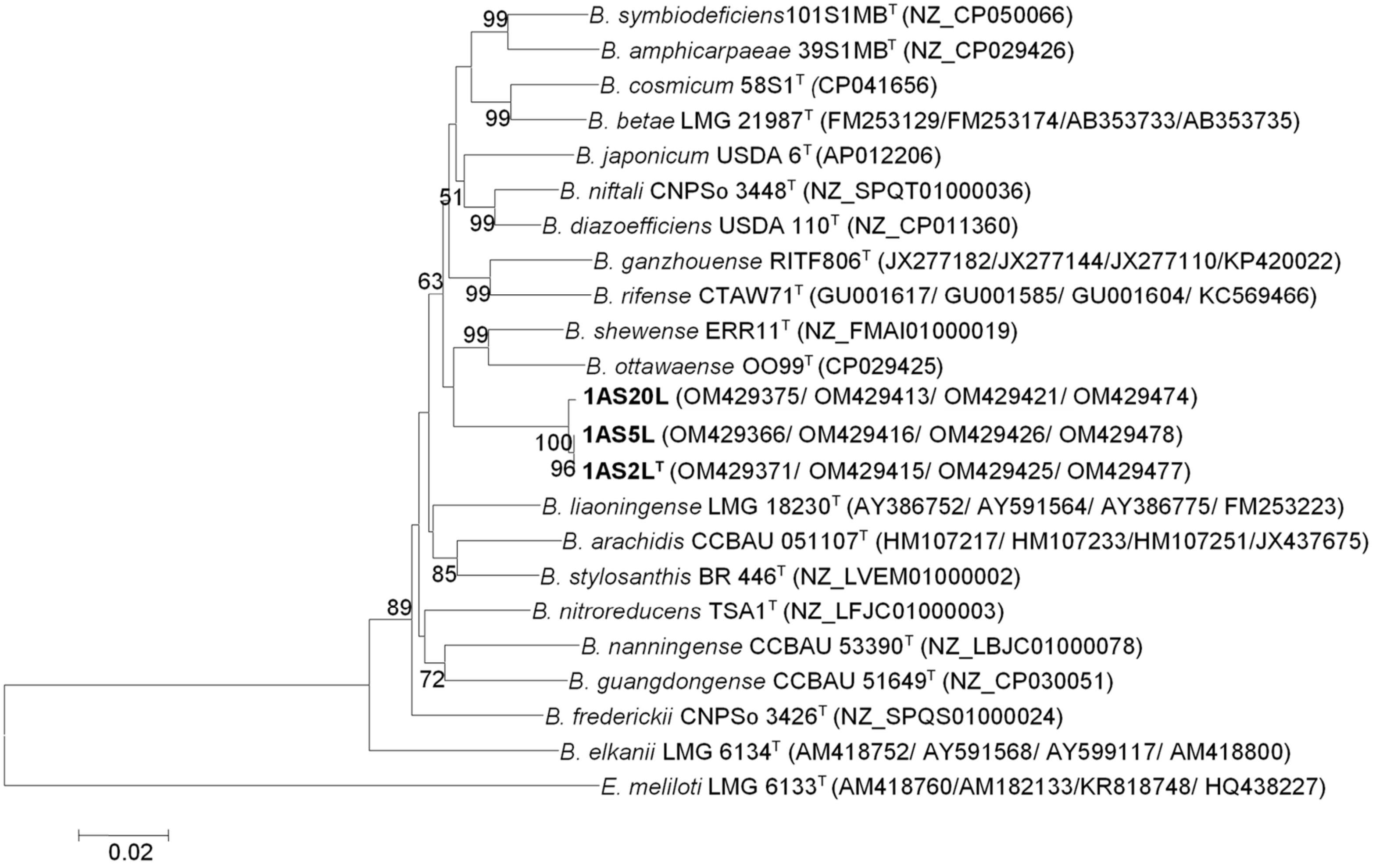

Figure S2. Maximum Likelihood-based phylogenetic analysis conducted using concatenated gene sequences of *atp*D, *rec*A, *gln*II, and *gyr*B (1,734 nucleotides). The three novel strains are indicated in bold. Bootstrap values ≥50, based on 1,000 replicates, are shown for each node. For each species, the strain designation and NCBI accession number of the sequence used are provided. The scale bar represents the number of substitutions per site.

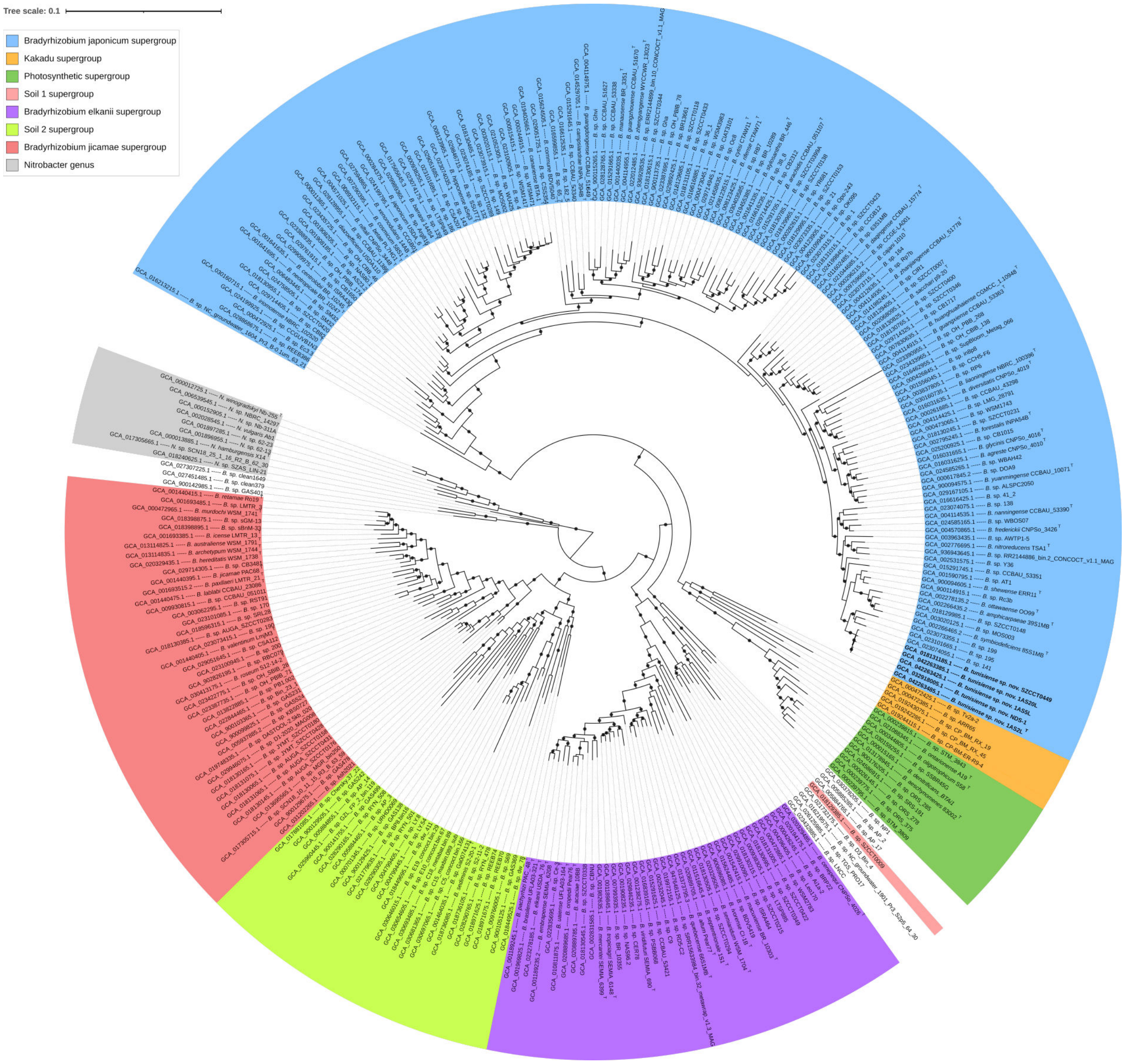

Fig. S3. De novo approximately-maximum-likelihood phylogenomic tree showing the seven supergroups of the genus Bradyrhizobium with the novel strains highlighted in bold. The tree was constructed using 117 bacterial gene markers (Table S2) as implemented in GTDB database reference genomes, and was rooted to the genus Nirobacter. Local support values >= 70 % are shown as cirles on the corresponding branches (based on 1000 resamples). Type strains are indicated by superscript T. An ANI threshould of 96% was used for strain identification. The scale bar represents the number of substitions per site.

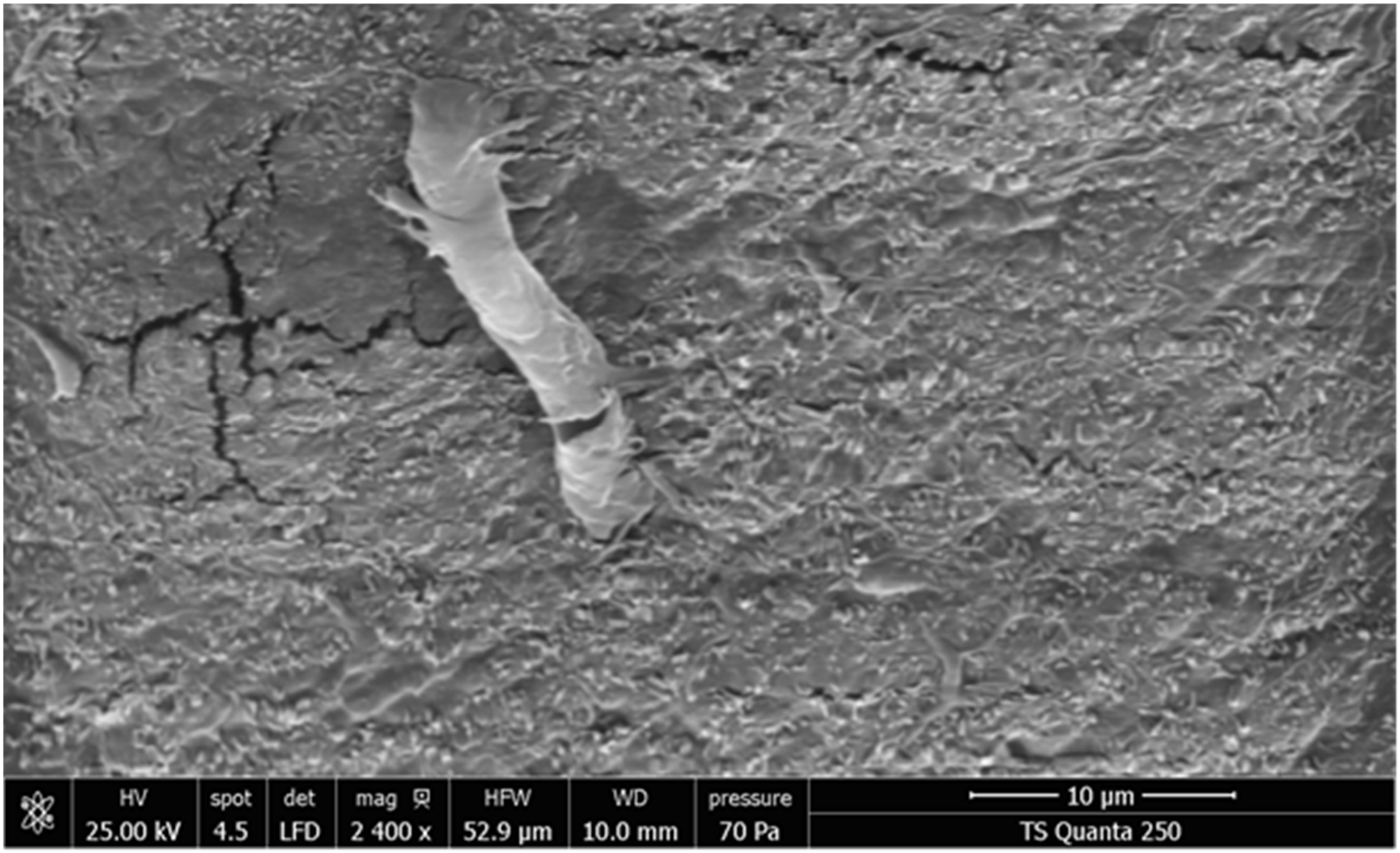

Fig. S4. Atomic force microscopy (AFM) images of the type strain 1AS11^{T} of *Bradyrhizobium tunisiense* sp. nov. showing its rod-shaped cell.
